# First U-Pb zircon ages for late Miocene Ashfall *Konservat-Lagerstätte* and Grove Lake ashes from eastern Great Plains, USA

**DOI:** 10.1371/journal.pone.0207103

**Published:** 2018-11-08

**Authors:** Jon J. Smith, Elijah Turner, Andreas Möller, R. M. Joeckel, Rick E. Otto

**Affiliations:** 1 Kansas Geological Survey, Lawrence, KS, United States of America; 2 The University of Kansas, Department of Geology, Lawrence, KS, United States of America; 3 Conservation and Survey Division, School of Natural Resources and Department of Earth and Atmospheric Sciences, University of Nebraska-Lincoln, Hardin Hall, Lincoln, NE, United States of America; 4 University of Nebraska–Lincoln, University of Nebraska State Museum, Lincoln, Nebraska, United States of America, and Ashfall Fossil Beds, University of Nebraska State Museum, Royal, Nebraska, United States of America; Royal Holloway University of London, UNITED KINGDOM

## Abstract

This paper documents the first U-Pb zircon ages for Ashfall Fossil Beds (Nebraska, USA), a terrestrial *Konservat-Lagerstätte* mass-death assemblage that is arguably the most diverse of its type and age. The Ashfall tephra was correlated with ignimbrites from the Bruneau-Jarbidge volcanic field (12.7–10.5 Ma) in southwest Idaho based on geochemical analysis. The methods and geochemical data supporting the original age assessment of the ash bed, however, were never published, and there has been a persistent misconception that dateable heavy minerals (e.g., zircon) are absent. Notwithstanding, we recovered abundant zircons from Ashfall Fossil Beds, and from an ash bed ~6 km to the southeast at Grove Lake, Nebraska, and analyzed them through LA-ICP-MS. Our new zircon U-Pb age of 11.86 ± 0.13 Ma substantiates correlation of the Ashfall Fossil Beds deposit to tuffs originating from the Bruneau-Jarbidge caldera (~12.7–10.5 Ma). Our U-Pb zircon age of 6.42 ± 0.06 Ma for the Grove Lake ash bed coincides with supervolcanic activity in the Heise volcanic field (6.6–4.3 Ma) in eastern Idaho. These new dates improve age constraints of strata comprising the Ogallala Group and the important paleontological site. Moreover, we find that detrital and airfall zircons are unevenly distributed in the stratified ash beds we describe herein and presumably in similar deposits worldwide. Therefore, a higher-resolution sampling scheme is necessary in such cases.

## Introduction

**Fig 1:**
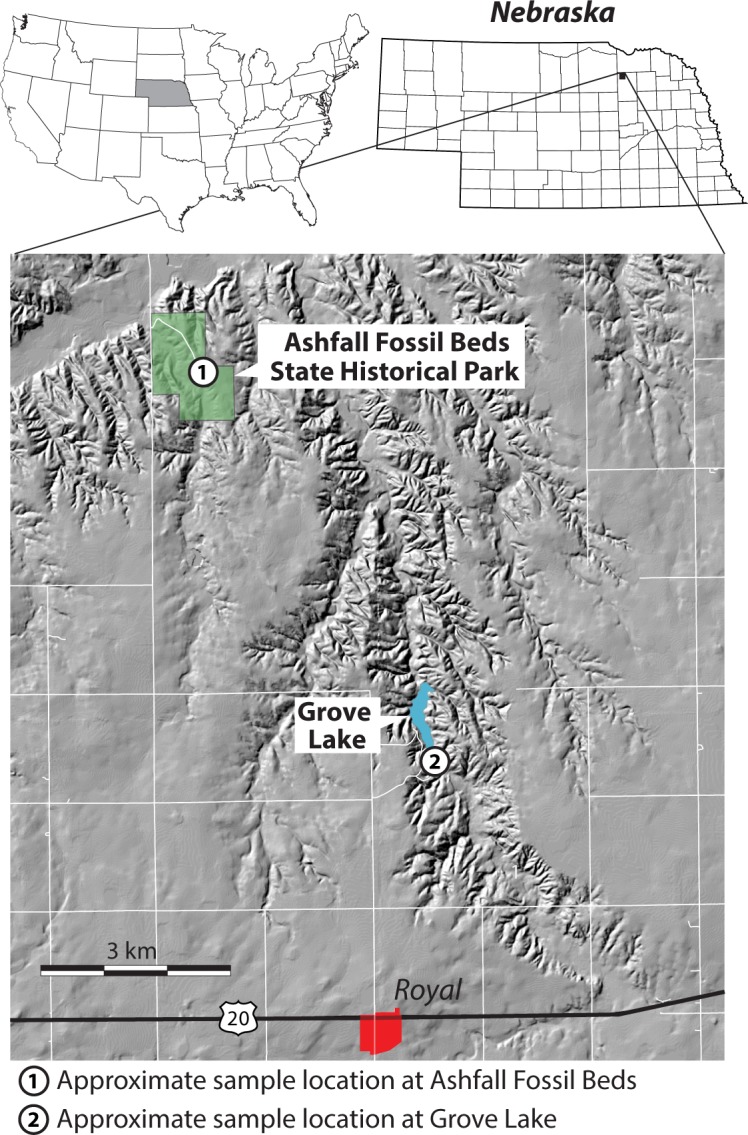
Location of study areas of Ashfall Fossil Beds State Historical Park and Grove Lake, Nebraska. Base map is DEM hillshade downloaded from the from USGS National Map Viewer (open access) at https://viewer.nationalmap.gov/viewer/.

Ashfall Fossil Beds State Historical Park (Fig 1), a U.S. National Natural Landmark, is a terrestrial *Konservat-Lagerstätte* mass-death assemblage composed of Miocene amphibians, reptiles, birds, and mammals within an ~3-m-thick vitric ash deposit [1, 2]. The area encompassing Ashfall Fossil Beds has been the focus of nearly continuous paleontological research since its discovery in the early 1950’s (e.g. [1, 2, 3, 4, 5, 6, 7, 8, 9, 10, 11]). In recent decades, Ashfall is frequently associated in the popular press (e.g. [12]) with the concerns regarding the scale and potential impacts of Yellowstone supervolcanic eruptions (ejecta volumes >100 km^3^) [13].

Ash beds, and K-bentonites as their weathered equivalents, are critical geochronological marker horizons because tephrochronologic correlations or the radiometric dating of volcanic minerals of airfall tephra constrain the depositional ages of host strata and are significant for the development of a regional chronostratigraphy (e.g. [14]). Tephrochronology is a geochemical technique that correlates distal ashfall deposits to well-dated proximal volcanic tuffs through the electron probe microprobe analysis of glass shards (e.g. [15, 16, 17]). Conventional wisdom maintains that preferred phenocrysts for the direct radiometric dating of ash beds, such as zircon and sanidine, are often rare or absent in distal airfall deposits [17].

Until the present paper, there have been no absolute ages published from Ashfall Fossil Beds. The bulk-glass chemistry of the ash correlates with ignimbrites of the Bruneau-Jarbidge volcanic field of the Snake River Plain in southeastern Idaho [18, 19, 20, 21]. This attribution is plausible because the *Lagerstätte* has been assigned to the Clarendonian North American Land Mammal Age, which spans 13.6 to 10.3 Ma [2, 22], and which overlaps the span of active volcanism (12.7−10.5 Ma) in the Bruneau-Jarbidge field [16]. Information regarding the tephrochronologic correlation of the Ashfall tephra to tuffs from the Bruneau-Jarbidge caldera, however, was disseminated primarily through personal communications (see [2]), conference presentations and abstracts [18, 19, 21], and popular media reporting (e.g. [12, 20, 23, 24, 25]). In no way do we criticize past researchers in underscoring these points. Rather, we amplify their work through a different approach to dating, for which we describe rigorous specific methods and data supporting tephrochronologic correlations, thereby certifying the age of the ash and its *Lagerstätte* with the first directly measured absolute age at the site.

We conducted a direct test of the age of the Ashfall *Lagerstätte* by collecting samples from four superposed stratigraphic intervals at Ashfall and another four from an ash bed cropping out ~6 km to the southeast at Grove Lake (Fig 1). Zircon, sanidine, and other minerals used for dating volcanoclastic sediments were hitherto assumed to be absent from the fine-grained vitric ash deposit [2]. Processing our samples with standard mineral separation techniques, however, yielded abundant zircon crystals in the lowermost sampled intervals at both locations. We dated these zircon crystals through laser ablation inductively coupled mass spectrometry (LA-ICP-MS) and obtained U-Pb ages of eruption. Ages of eruption are identical to deposition within the limits of reproducibility of the technique (1–2%) [26]. Our new U-Pb ages are a significant step in the development of a chronostratigraphic framework of Cenozoic deposits in the Great Plains of North America. Furthermore, our research indicates that detrital and airfall zircons are not distributed evenly in stratified ash beds, particularly in reworked deposits, and that finding such grains may require a higher-resolution sampling scheme than is typically used.

## Geologic background

**Fig 2:**
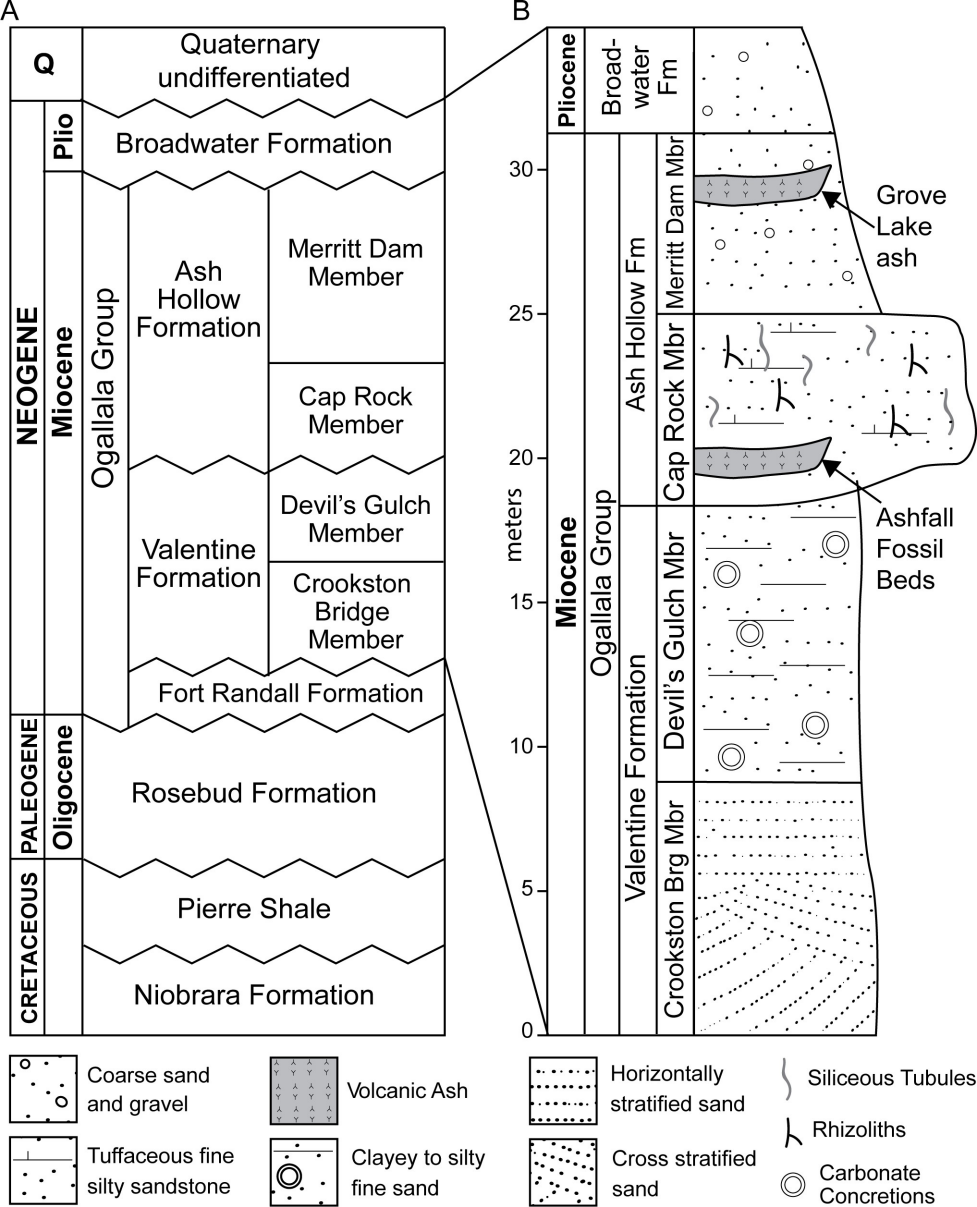
Stratigraphy of northern Antelope County, Nebraska. (A) Distribution of Cretaceous and Cenozoic strata in northeastern Nebraska. (B) Composite stratigraphic section of units exposed at the Ashfall site and Grove Lake. Modified from Voorhies [1] and Tucker et al. [2].

The ash deposits exposed at Ashfall Fossil Beds State Historical Park and Grove Lake are both within the Miocene Ash Hollow Formation of the Ogallala Group (Fig 2) [2, 27, 28]. The Ogallala Group (Ogallala Formation outside of Nebraska) underlies much of the North American High Plains and consists chiefly of fluvial sand, sandstone, silt, and siltstone, with minor eolian sediments and local lenses of volcanic ash, and lacustrine limestones and diatomites [29, 30]. The Ogallala Group is composed of several formations in Nebraska [31, 32], but only the Valentine and Ash Hollow Formations, which comprise most of it, are recognized in the immediate vicinity of Ashfall Fossil Beds [23, 31, 33, 34, 35]. The Ashfall *Konservat-Lagerstätte*, is in the Cap Rock Member of the Ash Hollow Formation. The ash bed exposed at the nearby Grove Lake lies within the overlying Merritt Dam Member of the same formation (Fig 2).

The ash bed at Ashfall Fossil Beds is interpreted as the fill of a small depression on the basis of its lenticular geometry and the taphonomy and paleoecology of the *Lagerstätte*. Indeed, many volcanic ash beds in continental successions are swale-filling deposits having limited lateral extents [17, 28]. The remains of fossil diatoms and such small aquatic vertebrates as salamanders, frogs, and turtles [2, 9], in addition to the high concentration of mammalian fossils, suggests that the Ashfall site was a transient watering hole at the time of ash deposition. Seasonally dry subtropical savannas extended across Nebraska in the Miocene with year-round above freezing temperatures, as indicated by the presence of large tortoises [36, 37]. We interpret the younger Grove Lake ash as a depression fill as well.

### Paleontology

**Fig 3:**
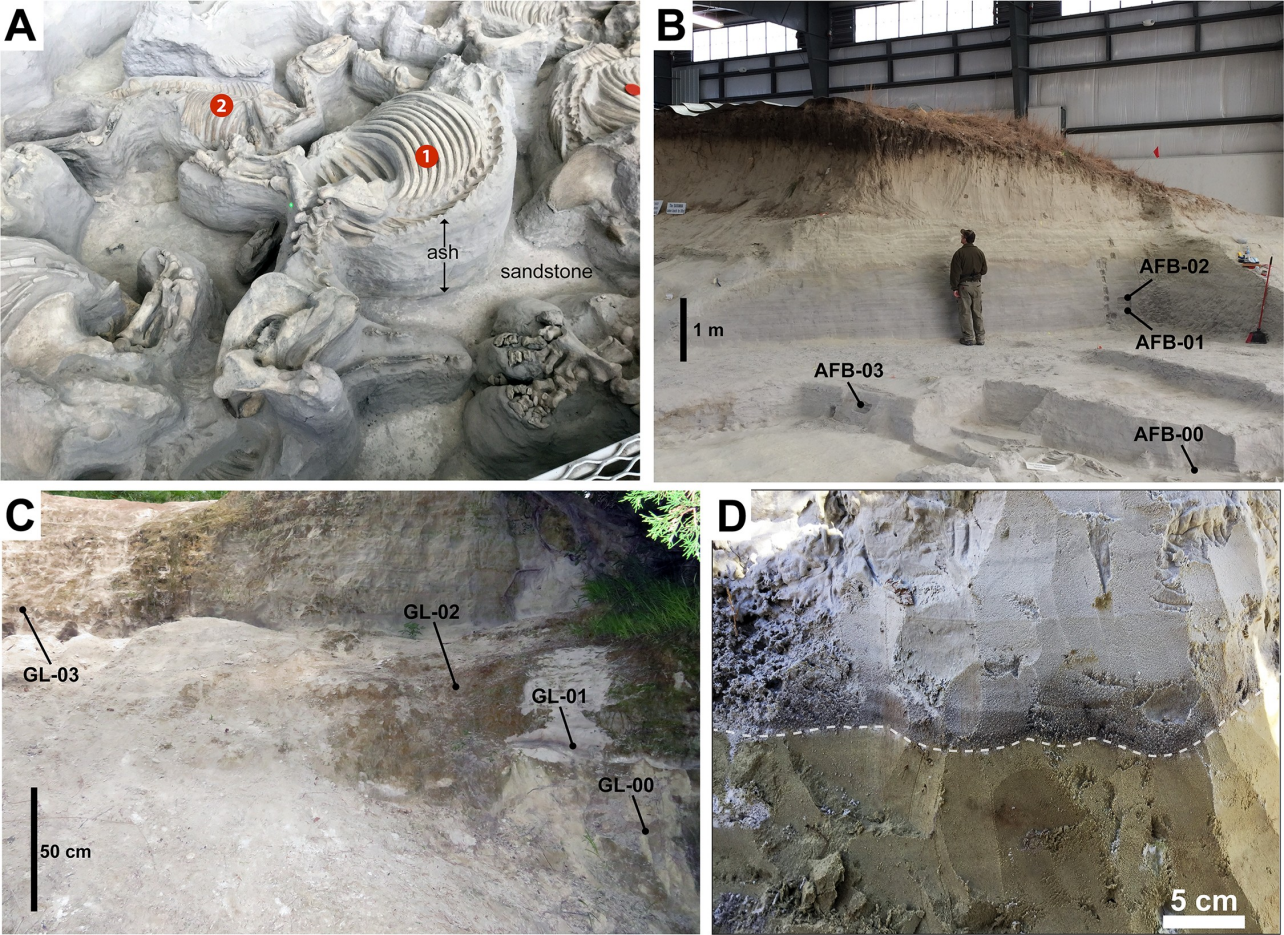
Outcrop photographs of the Ashfall Fossil Beds and Grove Lake ash localities. (A) Ashfall *Konservat-Lagerstätte* from inside the Hubbard Rhino Barn showing ash pillars supporting (1) fully articulated fossils of Teleoceras major and (2) underlying medium-sized equid. (B) Ash bed in the Cap Rock Member exposed in the Hubbard Rhino Barn at Ashfall Fossil Beds showing sample locations for this study. (C) Ash bed in the Merritt Dam Member just west of Grove Lake and sample locations for this study. (D) Photograph of the dark colored basal horizon at Ashfall that contains dateable volcanogenic zircons (immediately above dotted line).

The Ashfall Fossil Beds contains an extraordinary terrestrial *Konservat-Lagerstätte* of Miocene vertebrates in a mass death assemblage (Fig 3) [6]. The *Lagerstätte* is composed of 21 fossil taxa representing amphibians, reptiles, birds, and mammals of the Clarendonian NALMA (13.6–10.3 Ma) in north-central Nebraska [2, 23]. The most numerous and well-known fossil remains are those of the barrel-bodied rhinoceros, *Teleoceras major* (Perissodactyla, Rhinocerotidae). Over 100 specimens of *T*. *major* have been uncovered in the tephra deposit, and it is hypothesized that most or all were members of a single social group [8]. Many of the medium- to large-sized mammal skeletons are fully articulated and preserved in their original three-dimensional death poses.

The mass mortality *Lagerstätte* was the direct result of volcanic ash deposition, though several taphonomic lines of evidence suggest that medium- to large-sized ungulates succumbed to long-term ash exposure, as opposed to a single catastrophic burial event [2]. The first line of evidence is that the fossil taxa within the *Lagerstätte* are distributed vertically into three distinct and superposed assemblages separated by ~10–15 cm of unfossiliferous ash (Fig 3A). The base of the ash bed contains the mostly fragmentary remains of such relatively small animals as turtles and wading birds. The second fossiliferous horizon contains medium-sized taxa, including several species of camelids and equids. The third assemblage, ~25 cm from the base of the ash bed, is composed mostly of fully articulated *Teleoceras major* skeletons. The vertical distribution of the fossil taxa suggests successive die-offs according to different body sizes and abilities to withstand exposure to the ash. The second line of evidence suggesting that the larger vertebrates survived the initial airfall event, at least in the short-term, is abnormal growths on the bones of equids, camelids, and rhinos that resemble hypertrophic osteopathy or Marie’s disease [7]. Hypertrophic osteopathy is a bone pathology in mammals secondary to severe pulmonary diseases and inhalation of foreign objects [38]. These growths indicate that the larger ungulates succumbed only after several weeks or months of exposure to the ash after the initial airfall event.

### Airfall tephra and tephrochronologic correlations

**Fig 4:**
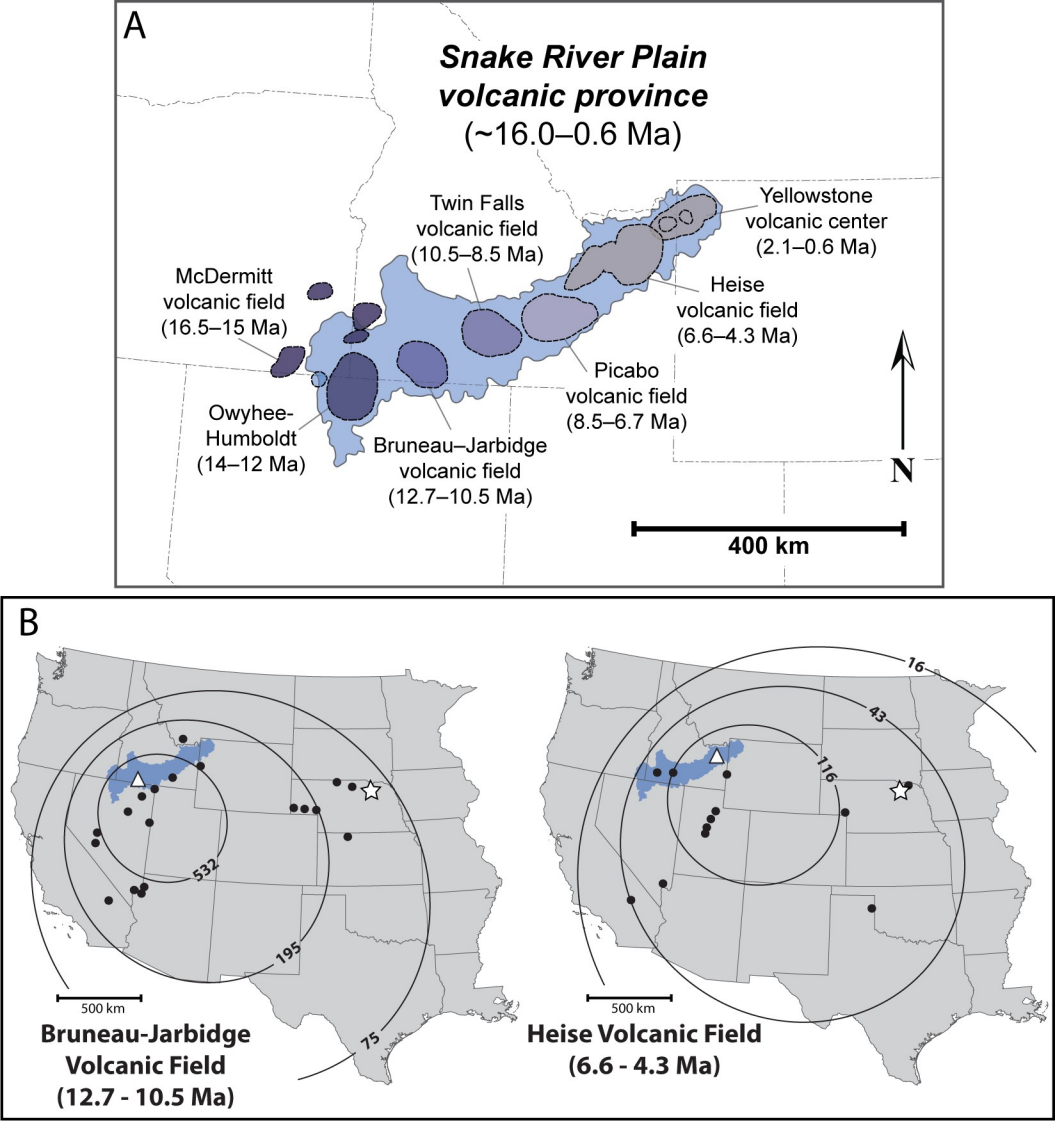
(A) The Snake River Plain Volcanic Province and its constituent volcanic fields and their age ranges of magmatic activity; modified from Perkins and Nash [16]. (B) Apparent accumulation rates (cm/m.y.) of ash beds from Bruneau-Jarbidge and Heise volcanic fields; modified from Perkins [43]. Note approximate locations of source calderas (white triangles), recognized airfall tuff deposits (black dots), and the Ashfall Fossil Beds and Grove Lake study area (white star). Base maps downloaded and modified from the USGS National Map Viewer (open access) at https://viewer.nationalmap.gov/viewer/.

The Snake River Plain is an ~770 km long, northeast-southwest trending linear depression that extends primarily across southern Idaho (Fig 4A) and contains seven major volcanic provinces that are progressively younger toward the northeast [39, 40, 41]. These include, from west to east, the McDermitt, Owyhee-Humboldt, Bruneau-Jarbidge, Twin Falls, Picabo, Heise, and Yellowstone Plateau volcanic fields [16]. Volcanism in this region began ca. 16.6 Ma near the Nevada-Oregon border with an outpouring of flood basalts on the Columbia Plateau and the formation of rhyolitic eruptive centers further south [16]. In Neogene strata of the Great Plains, volcanic ashes from the Snake River Plain volcanic province are preserved as airfall tephra composed primarily of fine-grained micron-scale rhyolitic glass bubble-wall shards sorted by long-distance atmospheric transport [42].

Proximal tuffs within the Bruneau-Jarbidge volcanic field range in age from ~12.7–10.5 Ma and are correlated regionally to distal airfall deposits in Idaho, Nevada, California, Colorado, New Mexico and throughout the Great Plains (Fig 4B) [16, 17, 43, 44, 45, 46, 47]. Total cumulative eruptive volume from Bruneau-Jarbidge is estimated to be 10^4^–14^4^ km^3^ [16, 48]. If an estimated 30% of this mass was dispersed as airfall ash [49], the largest individual eruption events at Bruneau-Jarbidge were potentially capable of producing over 1000 km^3^ of distal airfall deposits [48].

In a published abstract, Perkins [19] tentatively correlated the Ashfall Fossil Beds tephra with a tuff within the Bruneau-Jarbidge volcanic field, the Ibex Hollow Tuff (11.93 Ma). According to Perkins and Nash (see fig 5 in [16])], at least 22 Miocene-age tuffs of the Snake River Plain can be tephrochronologically correlated to ashes on the Great Plains, though the authors did not report the locations and stratigraphic positions of these distal ashes. Dobbins [50] made a tentative geochemical correlation of the Ashfall deposit to a Bruneau-Jarbidge eruption, the 10.45 ± 0.10 Ma Cougar Point Tuff XV, and concluded that the Ashfall deposit likely resulted from a single eruption event.

**Fig 5:**
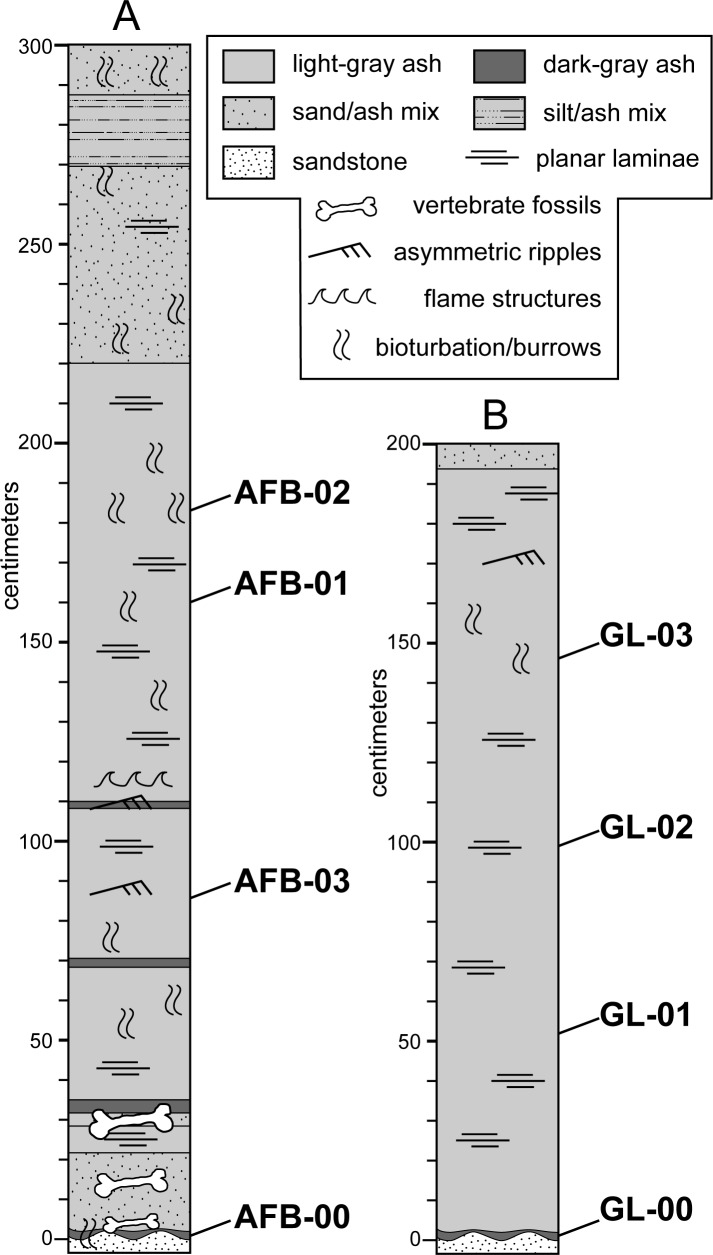
Measured sections of ash bed deposits in this study. (A) Ashfall Fossil Beds and (B) Grove Lake sections showing stratigraphic location of samples (AFB and GL, respectively). Some details of Ashfall Fossil Beds measured section (A) after Scheel and Rogers [10].

## Materials and methods

We collected eight bulk sediment samples from ash beds located at Ashfall Fossil Beds (42.420, -98.156) and nearby Grove Lake (42.375, -98.119) for analysis. We obtained permission to access and sample study co-author, Rick E. Otto, park superintendent of Ashfall Fossil Beds State Historical Park. Four volcanic ash samples were collected inside the Hubbard Rhino Barn at Ashfall Fossil Beds State Historical Park (Fig 3B). A second set of four ash samples was collected approximately 6 km southeast of Ashfall Fossil Beds from an ash bed near Grove Lake (Fig 3C). At both locations, approximately 4-liters of ash were collected from four discrete stratigraphic intervals (Fig 5A and 5B). The outermost 5–8 cm of the outcrop was scraped away prior to sample collection in order to maximize sample quality and avoid contamination with younger material washed down on the exposed face of the outcrop. At both locations, the first samples were taken from the bottom ~1–2 cm of the ash above the basal contact, which contained coarser-grained material and was darker gray than the overlying ash (Fig 3D). We moved up-section for the three remaining samples at each site, taking one sample approximately every 50–80 cm (Fig 5).

We isolated zircons from the ash samples using standard mineral separation techniques at the University of Kansas Isotope Geochemistry Laboratories (IGL). Each 1-liter sample was processed with an ultrasonic clay separator (UCS), using the methods described by Hoke et al. [51], to separate high-density minerals. After drying, 150g of the high-density split of each sample was slowly added to a polytetrafluoroethylene (Teflon) container containing 12 N HF to dissolve the SiO_2_-rich volcanic glass component of the ash. Finally, the remaining sample was again subjected to density separation, this time using heavy liquids (methylene iodide). Using a binocular microscope, zircon grains were handpicked from the high-density heavy liquid separates and mounted on double-sided adhesive tape on 1-inch diameter epoxy-resin discs. The grains were not polished to expose their interiors, in order to date the latest phase of zircon crystallization just prior to, or during, volcanic eruption [52].

Zircon U–Pb ages were obtained by laser ablation inductively coupled mass spectrometry (LA-ICP-MS) using a Thermo Scientific Element2 ICP-MS, attached to a Photon Machines Analyte.G2 193 nm ArF excimer laser ablation system. 20 μm circular spots were ablated with the laser at 2.0 J cm-2 fluence and 10 Hz repetition rate, resulting in pits of ~15 μm in depth from the surface towards the interior of each grain. Where feasible, the ablation targeted the tips of grains to avoid potentially older interiors an approach used in other studies (e.g. [53]). The ablated material was carried to the ICP-MS in He gas with a flow rate of 1.1 l/min, tied in with Ar gas of 1.1 l/min flow rate ca. 25 cm before entry into the plasma torch. Laser-induced fractionation, including elemental fractionation and downhole fractionation, and calibration drift were corrected by bracketing measurements of unknowns with the 608.5 ± 0.4 Ma GJ1 zircon reference material [54] and data reduction using the VizualAge data reduction scheme [55] for the IOLITE software package [56, 57]. Uncertainty (±2σ) in ^206^Pb/^238^U dates from individual GJ-1 ablations is approximately 8 Ma, uncertainties on larger data populations are at 1–2%. Calibration accuracy was monitored by measurement of zircon reference materials of known age during the same sessions, the Plešovice zircon [58] and Fish Canyon Tuff zircon [59], which were reproduced to within 1% of their published ages.

Our analytical results are presented using Isoplot 4.15 [60]. Kernel density estimates (KDEs) were produced using DensityPlotter 7.3 [61]. Eruption and maximum deposition ages (MDAs) of the volcanic ash deposits were derived from the youngest and most consistent grouping of concordant grains with overlapping U-Pb ages at 2σ. Because the Ashfall and Grove Lake samples contain large numbers of dates overlapping within 2 standard error (SE) uncertainties, we calculated the ages from analyses with a MSWD of 1 on the weighted average ^206^Pb/^238^U age (i.e. the scatter between analyses is equal to that expected for this number of analyses within a normal distribution). This is different from the approach to use the youngest three dates with overlapping uncertainties of Dickinson and Gehrels [62] proposed specifically for detrital populations that may contain only very few zircons close to the MDA. The rationale for our approach follows three main arguments. First, the measured dates represent zircon crystallization during the eruption event, or more likely, within the magma chamber before eruption. Crystallization within the magma chamber may have occurred over a time span longer than the uncertainty of the age determination, so including more analyses than expected from a normal distribution. Second, the calculated zircon age must be compared to Ar-Ar dates for these eruptions from the literature, which likely represent minimum (cooling) ages and are not influenced by "magma chamber inheritance". Including older zircons (MSWD >1) that may represent antecrystic zircon—crystals forming in the magma chamber significantly prior to the eruption (for a discussion of this term see e.g. [63, 64])—that will skew the calculated ages towards magma chamber process ages or even inherited earlier events. Third, extracting the age from only the youngest single analysis or very small group of analyses may bias the age determination towards a "tail" of the uncertainty distribution that is younger than the geological event and just represents statistical scatter. Hence the exclusion of a single concordant analysis from the interpretation of the Grove lake dataset. Analytical results are shown in S1 Table in Supporting Information.

## Results

### Ashfall Fossil Beds

**Fig 6:**
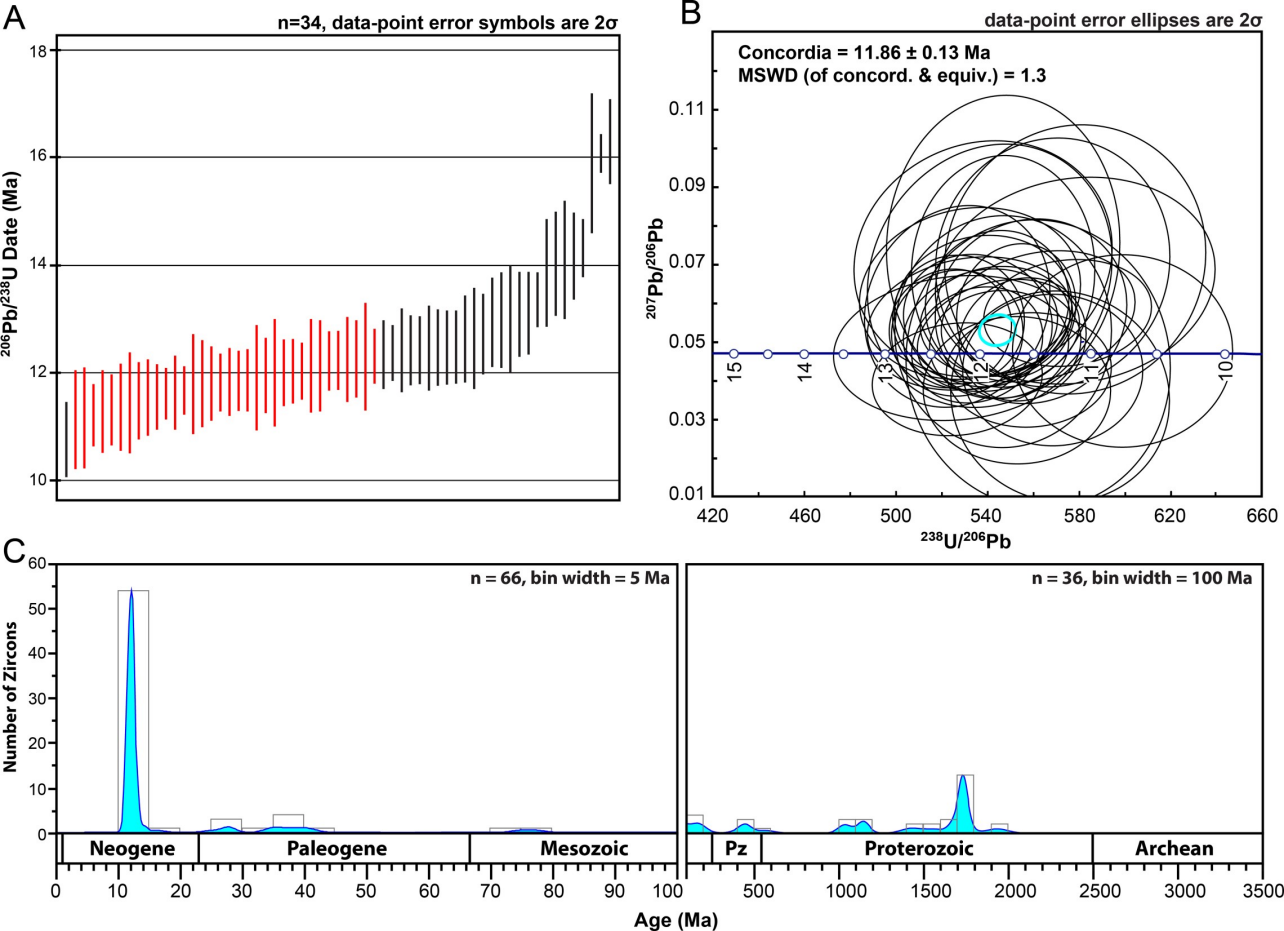
Zircon U-Pb results for the AFB-00 sample from Ashfall Fossil Beds. (A) U-Pb age ranges with 2σ error bars of the 61 youngest zircon grains plotted on a time axis showing 34 concordant analyses with an MSWD of ~1 (in red), used to calculate the MDA. (B) Tera-Wasserburg concordia diagram with MDA result of 11.86±0.13 Ma from 34 concordant analyses shown in 6A. (C) Kernel density estimate plot of AFB-00 showing ^206^Pb/^238^U (< 900 Ma) and ^207^Pb/^206^Pb (> 900 Ma) ages. MSWD = mean square weighted deviation; n = number of grains analyzed.

At Ashfall Fossil Beds, two samples yielded zircons during mineral separation: AFB-00 and AFB-01 (Fig 5A). AFB-00 was collected from a ~2 cm-thick horizon at the base of the ash that is relatively coarse grained and darker gray than to the rest of the deposit (Fig 3D). AFB-01 was collected ~160 cm up section from the base were the deposit is light gray and a nearly pure vitric ash (Fig 3B). Only 5 zircon grains were recovered from AFB-01 and proved to be older, detrital grains that were therefore not used in the interpretation of the depositional age for the ash. AFB-00 yielded 136 zircon grains and the youngest concordant grouping of zircons with overlapping ages at 2σ is composed of 34 grains yielding a concordia age of 11.86 ± 0.13 Ma with a MSWD of 1.3 (Fig 6A and 6B). This is interpreted as a crystallization age and the maximum depositional age (MDA) of subsequent volcaniclastic deposition. A Kernel density plot of U-Pb ages from AFB-00 (Fig 6C) shows that while the largest and youngest age population is middle Miocene, there are smaller, yet significant, age populations dating back to ~1980 Ma; suggesting detrital input from extrabasinal detrital-source areas, rather than mineral fractions contributed by tephra.

### Grove Lake

**Fig 7:**
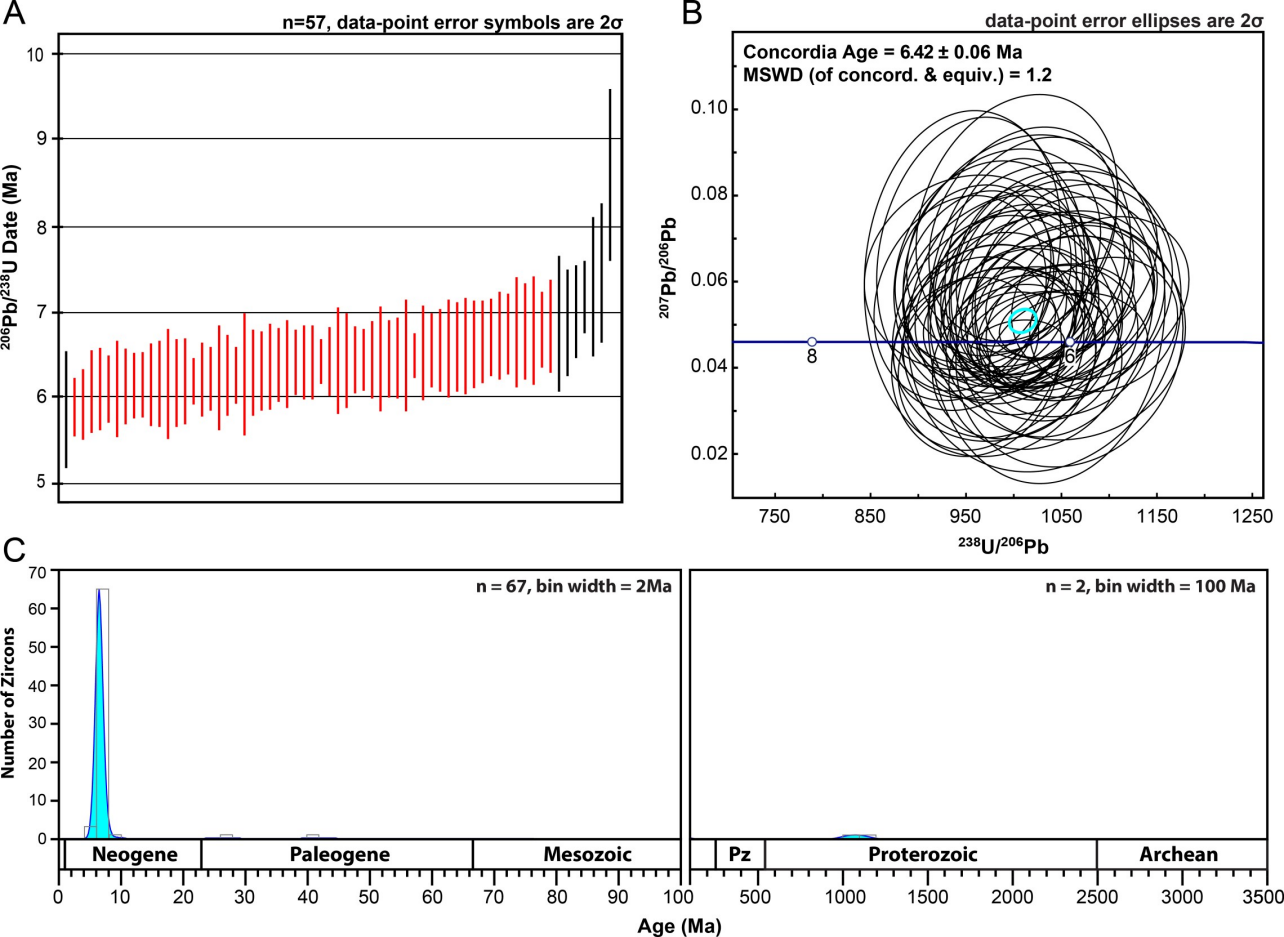
Zircon U-Pb results for the GL-00 sample from Grove Lake. (A) U-Pb ages ranges with 2σ error bars of the 65 youngest zircon grains plotted on a time axis showing 57 concordant analyses with an MSWD of 1 (in red), used to calculate the MDA. (B) Tera-Wasserburg concordia diagram with MDA result of 6.42±0.06 Ma from the 57 analyses with an MSWD of ~1 from 7A. (C) Kernel density plot of all GL-00 concordant results showing ^206^Pb/^238^U (< 900 Ma) and ^207^Pb/^206^Pb (> 900 Ma) ages. MSWD = mean square weighted deviation; n = number of grains analyzed.

At Grove Lake, GL-00 was the only sample that yielded zircons during mineral separation (Fig 5B). As in AFB-00, sample GL-00 was collected from a ~2 cm-thick horizon at the base of the ash that is relatively coarse grained and darker gray than the rest of the overlying deposit (Fig 3C). In total, 102 grains from GL-00 were analyzed. Fifty-seven of the youngest concordant grains yield a weighted average ^206^Pb/^238^U age of 6.36 ± 0.06 with a MSWD of 1.0, and were used to calculate a concordia age of 6.42 ± 0.06 with a MSWD of 1.18 and interpreted as the crystallization age (Fig 7A and 7B). A Kernel density plot of U-Pb ages from GL-00 shows that the largest age population is Late Miocene in age (Fig 7C). Smaller grain populations as old as ~1150 Ma suggest some detrital input, though less so than in the Ashfall Fossil beds deposit.

## Discussion

### Ash sources

**Fig 8:**
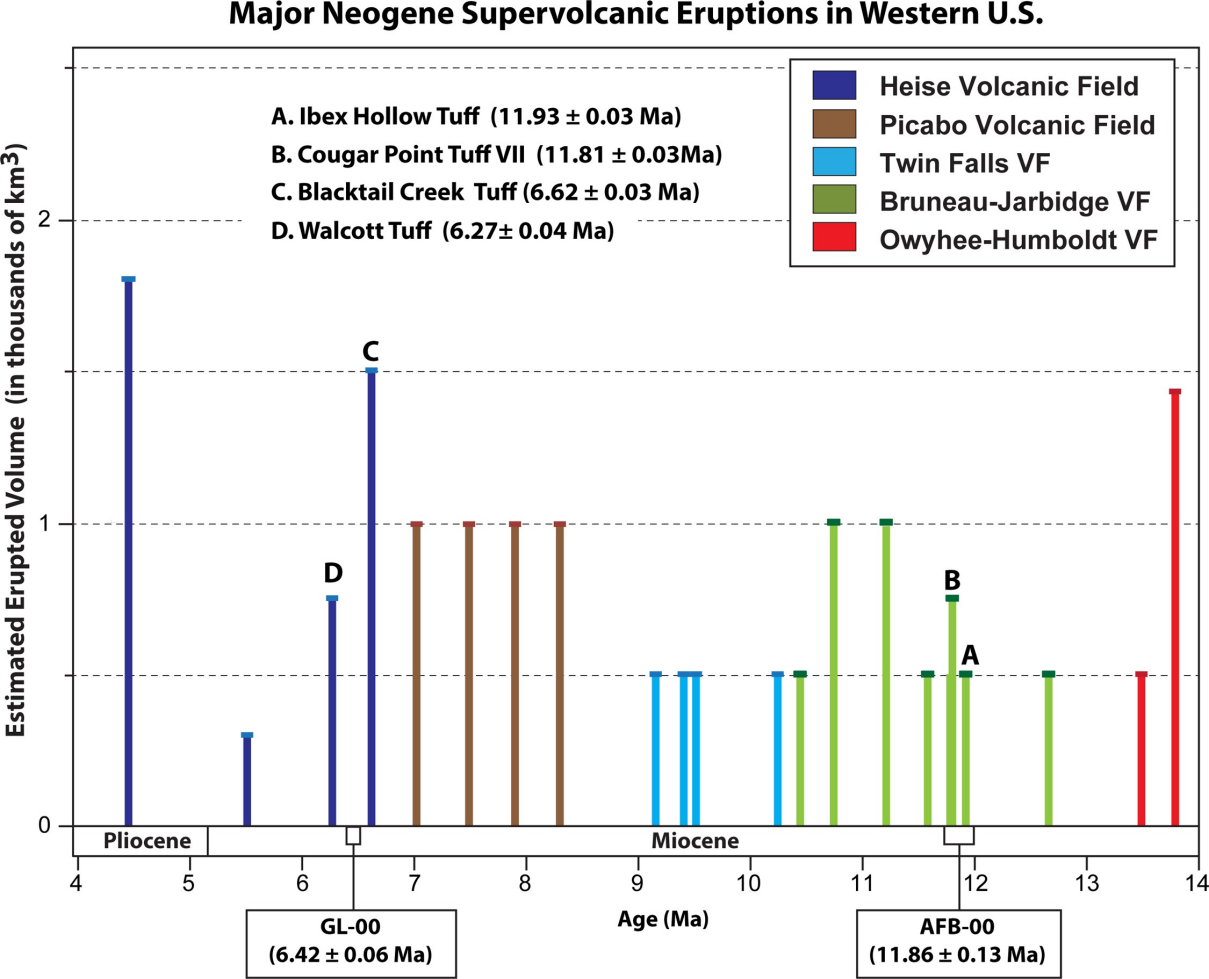
Ages and estimated volumes of known supervolcanic eruptions (ejecta volumes > 100 km^3^) from the Snake River Plain hotspot and reportedly correlated with ash beds on the Great Plains. Eruption ages and ejecta volumes (estimated with large uncertainties; [49]) were derived from tuffs proximal to source calderas [15, 16, 48]. Colors indicate individual volcanic fields of origin, letters denote dated tuffs discussed in the text that fall within or are close in age with the U-Pb age ranges for sample AFB-00 and GL-00. See S2 Table in Supporting Information for the list of supervolcanic eruptions constituting this figure, modified and updated from Smith et al. [78].

The zircon U-Pb age of 11.86 ± 0.13 Ma for the Ashfall Fossil Beds ash sample (AFB-00) is too old to support correlation with the 10.45 ± 0.10 Ma age interpolated for the Cougar Point Tuff XV [15], as suggested by Dobbins [50] on the basis of major and trace element compositions. Our results do corroborate the age correlation of the Ashfall ash to supervolcanic eruptions originating from the Bruneau-Jarbidge caldera from ~12.7–10.5 Ma in southwestern Idaho (Fig 4) [16]. Specifically, the Ashfall U-Pb age chronologically overlaps with the ^40^Ar/^39^Ar ages of the Ibex Hollow and Cougar Point VII ignimbrites at Bruneau-Jarbidge (Fig 8).

The Ibex Hollow Tuff is an ~4 m thick, rhyolitic, nonwelded tuffs first described from the Trapper Creek area in south-central Idaho and initially referred to as the Tuff of Ibex Peak (tuff 30 in [65]). It was originally reported to have an ^40^Ar/^39^Ar age of 11.81 ± 0.03 Ma on sanidine (>0.5 mm), obtained by laser fusion [65]. After recalibration to the Fish Canyon Tuff standard, this age was later adjusted to 11.93 ± 0.03 Ma [15, 16]. The Cougar Point Tuff (CPT) VII is one of nine densely welded ignimbrites originating from the Bruneau-Jarbidge caldera that are best exposed in canyons along the Bruneau and Jarbidge rivers in southwestern Idaho [66]. Bonnichsen et al. [48] reported an ^40^Ar/^39^Ar age of 11.81 ± 0.03 Ma from sanidine phenocrysts. Perkins et al. [15] identified two additional ash fall tuffs from southwest Idaho that are within the AFB-00 age range; the Ibex Peak-8 (11.80 ± 0.04 Ma) and Logan Ranch (^40^Ar/^39^Ar age of 11.79 ± 0.10 Ma) tuffs. Due to similarities in geochemical composition, stratigraphic position, and overlapping ages, however, Nash and Perkins [67] suspect that these ash beds are likely airfall equivalents of the CPT VII eruption event. Tephrochronologically correlated airfall equivalents of the Ibex Hollow and CPT VII tuffs are reported in multiple outcrops and cores from the Basin and Range province (Nevada and California), and the central Great Plains [16].

The U-Pb age of 6.42 ± 0.06 Ma for the Grove Lake ash (GL-00) coincides with supervolcanic activity in the Heise volcanic field (6.6–4.3 Ma) of the Snake River Plain in eastern Idaho (Fig 4). Our zircon-age is chronologically between, but does not overlap, with the ^40^Ar/^39^Ar ages of two well-documented ignimbrites of the Heise caldera; the Blacktail Creek and the Walcott tuffs (Fig 8). The Blacktail Creek Tuff is the most extensive (>12^4^ km^2^) welded ignimbrites of the Heise volcanic field [68] and has a mean ^40^Ar/^39^Ar age of 6.62 ± 0.03 Ma obtained from sanidine phenocrysts [69]. Morgan and McIntosh [69] geochemically correlate an airfall tuff with a younger ^40^Ar/^39^Ar age of 6.54 ± 0.06 Ma in the Palisades Reservoir area of Idaho. The smaller Walcott Tuff is a rhyolitic, welded ignimbrite that has a mean ^40^Ar/^39^Ar age of 6.27 ± 0.04 Ma also obtained from sanidine [69]. Blacktail Creek and Walcott tuffs have been tephrochronologically correlated to airfall ash beds in Idaho, Montana, Utah and the Great Plains region [16].

We consider our results as confirmation of the previously reported tephrochronologic correlations of the Ashfall Fossil Beds ash and Grove Lake ash to the Bruneau-Jarbidge and Heise volcanic fields, respectively. Correlating the Nebraskan ashes with specific ^40^Ar/^39^Ar-dated Snake River Plain ignimbrites and airfall tuffs, however, is complicated by several factors. The ^40^Ar/^39^Ar ages of the various proximal ignimbrites near Bruneau-Jarbidge are statistically indistinguishable from our ^206^Pb/^238^U zircon crystallization ages at the 2σ level. In the case of the Grove Lake ash, our U-Pb age is outside the uncertainty ranges of Heise ignimbrites, though the Grove Lake age does overlap with ^40^Ar/^39^Ar ages of airfall tuffs near the Heise caldera [69]. Perceived age discrepancies could be the result of different crystallization histories of the different dated phenocrysts prior to or during the eruption phase and the lack of congruence between ^206^Pb/^238^U and ^40^Ar/^39^Ar geochronometers (see [70]). Geochronological studies on independently dated volcanic eruptions have shown that zircon crystallization ages may predate eruption by several thousands of years [e.g. 71]. Although it is beyond the scope of this paper, recent efforts inter-calibrate the U-Pb and ^40^Ar/^39^Ar dating series are reported for different systems in Sageman et al. [70], and Trujillo and Kowallis [72].

### Implications of stratigraphic sampling of ash beds

Previous attempts to isolate zircons from the tephra at Ashfall were reportedly unsuccessful and it was assumed that radiogenic volcanoclastic material needed to date directly the *Konservat-Lagerstätte* was absent [2]. Given the lack of zircons throughout most of the 3 m-thick ash, it is evident that sampling from the ~1–2 cm horizon at the base of the ash was necessary in order to yield enough zircons for LA-ICP-MS analysis. If any previous sampling strategies overlooked this thin basal interval, it is indeed unlikely that such sampling would have yielded zircons indicative of the time of ash deposition. Rather, such hypothetical sampling would have yielded a low number of older detrital zircons, if any.

There are several *a priori* explanations for the concentration of zircons at the base of the ash bed, which include: 1) sorting during air transport, 2) heavier components of the ash cloud (e.g. zircons) precipitating to the ground before lighter components (e.g. glass, sanidine), and 3) internal stratification resulting from post-depositional reworking. Because of the lenticular morphology of the Ashfall ash (relatively thick in the immediate vicinity of the *Lagerstätte* and substantially thinner away from the *Lagerstätte*), it is interpreted to have infilled a paleodepression with ephemeral standing water [2]. Sedimentary structures such as asymmetrical ripples, planar laminae and flame structures suggest the presence of water in the paleodepression during ash accumulation. These structures in superposed ash intervals, as well as to multiple intervals of sediment bioturbation, suggest that the Ashfall ash bed is the product of a series of episodic depositional events likely separated by brief periods of subaerial exposure [10]. Furthermore, the presence of rounded, relatively old and large (up to ~100μm) detrital zircons indicates that the primary airfall deposit was mixed with sediment grains of other origins prior to final deposition at the site (Fig 6C). These pre-Neogene detrital zircons were likely derived from older deposits of the Ogallala Group. These lines of evidence indicate that the ash was reworked by water to some degree before infilling the topographic low at the Ashfall site.

Pyroclastic-fall deposits in submarine environments display a form of graded bedding characterized by an upward decrease in pyroclast density (e.g. [73, 74, 75, 76, 77]). If the ancient watering hole at all resembled a submarine environment in terms of depositional energy, this observation could explain the internal stratification seen in the Ashfall ash. The abundance of zircons in the lowest interval suggests that high density and/or coarser particles concentrated there via particle settling while the paleodepression was filled with a mixture of ash and water. Nevertheless, this pattern does not preclude sorting during air transport or prior, upwind precipitation of heavy minerals from the ash; these factors may also explain the observed upward decrease in density. In comparison, the ash bed at Grove Lake exhibits a similar style of internal stratification, but the dearth of information on the sedimentology of this ash hampers additional interpretation.

## Conclusions

Studies using the direct radiometric dating and tephrochronology of airfall tephra constraint depositional ages of host strata and are vital for the development of a regional chronostratigraphic framework on the Great Plains. We report new U-Pb zircon ages for the Ashfall Fossil Beds and Grove Lake ashes of 11.86 ± 0.13 Ma and 6.42 ± 0.06, respectively. These analyses provide the first absolute age constraint for the Ashfall *Konservat-Lagerstätte* and are a direct test of reported, though not well- substantiated tephrochronologic studies correlating Ashfall with tephra of the Bruneau-Jarbidge volcanic field in the Snake River Plain Volcanic Province. However, age correlating the Ashfall and Grove Lake ashes with specific proximal ignimbrites and airfall tuffs for which ^40^Ar/^39^Ar ages are known is complicated by several factors. These factors include the range of uncertainty within the U-Pb results themselves, and the lack of congruence between the ^206^Pb/^238^U and ^40^Ar/^39^Ar geochronometers. We plan to reduce the uncertainty and refine U-Pb zircon ages from Ashfall and Grove Lake using chemical abrasion isotope dilution thermal ionization mass spectrometry (CA-ID-TIMS), which uses dissolution of whole grains or parts of grains to obtain high-precision dates.

Our results also provide an admonition that applies to similar ash deposits of any age elsewhere. We opine that there should be no *a priori* expectation of uniform stratigraphic distribution of detrital and airfall zircons within ash bed deposits, particularly those showing evidence of being reworked. Only the lowermost ash layers in such a deposit can be expected to yield significant numbers of volcanic zircon. Thus, a closer examination of the distribution of zircons and other radiogenic mineral grains within airfall tephra deposits will likely determine the efficacy of different sampling strategies.

## Supporting information

S1 Table Zircon LA-ICP-MS U-Pb isotopic data used to calculate eruption ages for the Ashfall Fossil Beds (AFB-00) and Grove Lake (GL-00) ashbeds, Nebraska, USA. (PDF)

S2 Table Ages and estimated volumes of known supervolcanic eruptions (ejecta volumes > 100 km3) from the Snake River Plain hotspot and reportedly correlated with ash beds on the Great Plains. (PDF)
