# Supplementary material for: First U-Pb zircon ages for late Miocene Ashfall Konservat-Lagerstätte and Grove Lake ashes from eastern Great Plains, USA
Source: PLoS One. 2018 Nov 8;13(11):e0207103. doi: 10.1371/journal.pone.0207103 (PMC6224108; doi:10.1371/journal.pone.0207103)
Supplement: S1 Table — (PDF) [file pone.0207103.s001.pdf]

S1 Table. Zircon LA-ICP-MS U-Pb Isotopic Data and Ages for Smith et al., First U-Pb Zircon Ages from Ashfall Fossil Beds (AFB-00) and Grove Lake (GL-00), Nebraska, USA

						Corrected Isotopic Ratios							Ages (Ma)[h]							
Sample ID	Grain #[a]	U[b] [ppm]	Th[b] [ppm]	Th/U	eU[c] [ppm]	$^{207}Pb/^{235}U$[d]	±2s[e]	$^{206}Pb/^{238}U$[d]	±2s[e]	Rho[f]	$^{207}Pb/^{206}Pb$[g]	±2s[e]	$^{207}Pb/^{235}U$	±2s	$^{206}Pb/^{238}U$	±2s	$^{207}Pb/^{206}Pb$	±2s	Disc. %[k]	Wtd. Disc.[l]
AFB-00	AFB_49	106.3	60.5	0.57	120.52	0.01	0.005	0.0017	0.0001	-0.24	0.057	0.024	11.9	5.0	10.8	0.7	40.0	690.0	-10.6	0.2
AFB-00	AFB_112	48.9	27.4	0.56	55.33	0.02	0.010	0.0017	0.0002	-0.11	0.107	0.050	18.2	9.9	10.9	1.0	-500.0	1000.0	-67.0	0.7
AFB-00	AFB_140	166.5	91.0	0.55	187.89	0.02	0.005	0.0017	0.0001	0.18	0.074	0.018	18.7	4.4	11.1	0.5	710.0	470.0	-68.5	1.7
AFB-00	AFB_133	94.8	40.8	0.43	104.39	0.02	0.007	0.0017	0.0001	-0.10	0.075	0.034	16.7	6.8	11.1	0.9	240.0	730.0	-50.0	0.8
AFB-00	AFB_77	125.7	65.0	0.52	140.98	0.02	0.005	0.0017	0.0002	-0.20	0.072	0.025	15.6	5.2	11.2	0.9	270.0	630.0	-39.8	0.9
AFB-00	AFB_42	156.3	107.3	0.69	181.52	0.01	0.003	0.0017	0.0001	-0.16	0.058	0.013	13.7	2.7	11.2	0.6	280.0	360.0	-22.2	0.9
AFB-00	AFB_128	98.6	60.1	0.61	112.72	0.01	0.005	0.0018	0.0001	-0.15	0.050	0.028	9.1	5.3	11.3	0.8	-540.0	680.0	19.3	-0.4
AFB-00	AFB_129	126.2	89.8	0.71	147.30	0.01	0.004	0.0018	0.0001	-0.10	0.045	0.018	10.3	3.9	11.3	0.7	-180.0	510.0	8.9	-0.3
AFB-00	AFB_105	103.3	61.2	0.59	117.68	0.02	0.006	0.0018	0.0001	0.00	0.070	0.028	16.8	6.4	11.4	0.8	350.0	700.0	-47.9	0.9
AFB-00	AFB_73	160.2	98.2	0.61	183.28	0.02	0.003	0.0018	0.0001	0.18	0.068	0.015	16.5	3.4	11.4	0.7	510.0	410.0	-44.4	1.5
AFB-00	AFB_99	76.6	36.2	0.47	85.11	0.01	0.008	0.0018	0.0002	-0.09	0.038	0.030	10.5	7.5	11.4	0.9	-290.0	720.0	8.2	-0.1
AFB-00	AFB_94	191.2	116.8	0.61	218.65	0.02	0.005	0.0018	0.0001	-0.03	0.107	0.023	24.2	5.0	11.5	0.7	1470.0	460.0	-111.4	2.6
AFB-00	AFB_143	226.7	134.6	0.59	258.33	0.01	0.003	0.0018	0.0001	0.04	0.059	0.014	13.9	3.3	11.5	0.7	270.0	410.0	-21.1	0.7
AFB-00	AFB_30	121.6	69.0	0.57	137.82	0.01	0.005	0.0018	0.0001	0.11	0.057	0.020	14.2	4.8	11.5	0.7	350.0	540.0	-23.1	0.6
AFB-00	AFB_127	142.1	73.7	0.52	159.42	0.01	0.003	0.0018	0.0001	-0.13	0.043	0.012	10.6	3.0	11.6	0.6	-240.0	400.0	8.2	-0.3
AFB-00	AFB_102	154.1	93.3	0.61	176.03	0.02	0.004	0.0018	0.0001	-0.02	0.081	0.017	20.9	4.1	11.6	0.6	960.0	410.0	-80.6	2.3
AFB-00	AFB_27	155.9	92.9	0.60	177.73	0.01	0.003	0.0018	0.0001	0.07	0.050	0.013	12.5	3.3	11.6	0.5	20.0	420.0	-7.7	0.3
AFB-00	AFB_114	114.4	68.7	0.60	130.54	0.01	0.004	0.0018	0.0001	-0.08	0.041	0.017	9.2	3.9	11.6	0.7	-370.0	480.0	20.9	-0.6
AFB-00	AFB_144	177.5	126.7	0.71	207.27	0.01	0.004	0.0018	0.0001	0.06	0.059	0.015	14.4	3.5	11.7	0.5	190.0	420.0	-23.4	0.8
AFB-00	AFB_139	213.0	157.0	0.74	249.90	0.02	0.008	0.0018	0.0001	-0.02	0.108	0.080	18.6	8.0	11.8	0.9	800.0	940.0	-57.8	0.9
AFB-00	AFB_55	91.4	39.8	0.44	100.75	0.01	0.006	0.0018	0.0001	0.14	0.056	0.028	13.0	6.2	11.8	0.8	-400.0	640.0	-10.2	0.2
AFB-00	AFB_56	129.7	73.3	0.57	146.93	0.02	0.006	0.0018	0.0001	-0.02	0.084	0.024	21.8	5.8	11.8	0.7	1030.0	530.0	-84.7	1.7
AFB-00	AFB_122	103.9	49.9	0.48	115.63	0.02	0.006	0.0018	0.0001	-0.35	0.075	0.028	17.0	6.2	11.8	0.7	80.0	660.0	-44.1	0.8
AFB-00	AFB_93	164.8	101.6	0.62	188.68	0.01	0.003	0.0018	0.0001	-0.10	0.053	0.012	12.4	2.9	11.8	0.5	330.0	370.0	-4.9	0.2
AFB-00	AFB_98	183.8	120.9	0.66	212.21	0.02	0.004	0.0018	0.0001	0.03	0.087	0.015	21.3	3.5	11.8	0.6	1070.0	340.0	-80.2	2.7
AFB-00	AFB_84	167.0	114.4	0.69	193.88	0.01	0.003	0.0018	0.0001	0.01	0.053	0.013	13.0	2.9	11.8	0.6	190.0	380.0	-9.9	0.4
AFB-00	AFB_67	158.0	99.6	0.63	181.41	0.02	0.004	0.0018	0.0001	0.12	0.067	0.016	15.1	3.9	11.9	0.6	390.0	440.0	-27.4	0.8
AFB-00	AFB_52	177.2	127.0	0.72	207.05	0.01	0.003	0.0018	0.0001	0.14	0.055	0.011	14.0	2.9	11.9	0.6	240.0	360.0	-18.0	0.7
AFB-00	AFB_8	87.4	67.2	0.77	103.19	0.02	0.007	0.0018	0.0002	-0.16	0.102	0.035	21.6	6.7	11.9	1.3	730.0	670.0	-81.5	1.4
AFB-00	AFB_80	91.0	47.7	0.52	102.21	0.02	0.007	0.0019	0.0002	0.07	0.074	0.031	16.7	6.9	11.9	1.0	-30.0	760.0	-40.2	0.7
AFB-00	AFB_92	141.7	79.8	0.56	160.45	0.01	0.004	0.0019	0.0001	-0.14	0.065	0.019	14.9	4.0	12.0	0.7	260.0	500.0	-24.7	0.7
AFB-00	AFB_14	104.1	65.9	0.63	119.59	0.02	0.006	0.0019	0.0002	-0.12	0.069	0.030	18.1	6.4	12.0	1.0	200.0	700.0	-50.8	1.0
AFB-00	AFB_104	175.2	111.3	0.64	201.36	0.01	0.003	0.0019	0.0001	0.04	0.052	0.012	13.6	3.2	12.0	0.6	-30.0	380.0	-13.3	0.5
AFB-00	AFB_32	150.0	79.9	0.53	168.78	0.01	0.004	0.0019	0.0001	0.27	0.058	0.014	14.6	3.8	12.0	0.6	120.0	420.0	-21.4	0.7
AFB-00	AFB_37	181.1	130.6	0.72	211.79	0.01	0.003	0.0019	0.0001	0.02	0.049	0.012	12.6	3.1	12.0	0.5	-10.0	390.0	-4.7	0.2
AFB-00	AFB_86	164.0	110.2	0.67	189.90	0.02	0.003	0.0019	0.0001	0.10	0.078	0.014	19.5	3.2	12.1	0.6	910.0	350.0	-61.4	2.3
AFB-00	AFB_70	201.0	136.0	0.68	232.96	0.02	0.005	0.0019	0.0001	-0.03	0.071	0.026	16.1	4.7	12.1	0.9	480.0	530.0	-32.7	0.8
AFB-00	AFB_107	161.0	96.1	0.60	183.58	0.01	0.004	0.0019	0.0001	0.11	0.057	0.017	14.1	3.6	12.1	0.9	110.0	470.0	-16.2	0.5
AFB-00	AFB_88	820.0	501.0	0.61	937.74	0.03	0.004	0.0019	0.0001	0.46	0.094	0.013	25.1	3.2	12.2	0.7	1430.0	260.0	-105.9	4.0
AFB-00	AFB_57	188.9	116.1	0.61	216.18	0.01	0.004	0.0019	0.0001	0.19	0.060	0.014	14.5	3.6	12.2	0.6	350.0	410.0	-18.7	0.6
AFB-00	AFB_53	171.3	103.9	0.61	195.72	0.01	0.004	0.0019	0.0001	-0.03	0.058	0.014	13.6	3.5	12.2	0.6	270.0	390.0	-11.2	0.4
AFB-00	AFB_111	89.4	37.9	0.42	98.31	0.02	0.006	0.0019	0.0001	0.05	0.060	0.022	15.3	5.5	12.3	0.8	100.0	560.0	-24.9	0.6
AFB-00	AFB_28	131.2	70.9	0.54	147.86	0.01	0.003	0.0019	0.0001	0.10	0.039	0.012	10.5	3.1	12.3	0.7	-160.0	400.0	14.4	-0.6
AFB-00	AFB_147	114.3	69.1	0.60	130.54	0.01	0.004	0.0019	0.0002	-0.02	0.051	0.017	12.6	4.0	12.3	1.0	350.0	490.0	-2.4	0.1
AFB-00	AFB_76	179.0	119.0	0.66	206.97	0.01	0.003	0.0019	0.0001	0.12	0.055	0.013	14.1	3.3	12.3	0.5	480.0	370.0	-14.5	0.5
AFB-00	AFB_71	155.8	94.5	0.61	178.01	0.02	0.005	0.0019	0.0001	0.19	0.062	0.020	16.7	5.0	12.3	0.6	380.0	540.0	-35.3	0.9
AFB-00	AFB_110	240.1	216.8	0.90	291.05	0.01	0.002	0.0019	0.0001	0.21	0.049	0.008	12.0	1.9	12.4	0.5	120.0	280.0	3.0	-0.2
AFB-00	AFB_17	90.6	54.8	0.60	103.48	0.01	0.005	0.0019	0.0001	0.06	0.053	0.019	14.0	4.7	12.4	0.8	-40.0	510.0	-12.7	0.3
AFB-00	AFB_108	134.2	92.7	0.69	155.98	0.01	0.003	0.0019	0.0001	0.07	0.057	0.014	13.8	3.4	12.4	0.7	250.0	420.0	-11.1	0.4
AFB-00	AFB_142	242.0	159.5	0.66	279.48	0.01	0.003	0.0019	0.0001	0.07	0.059	0.012	14.7	3.0	12.4	0.6	460.0	350.0	-18.4	0.8
AFB-00	AFB_6	114.0	73.1	0.64	131.18	0.03	0.006	0.0019	0.0001	0.16	0.101	0.023	24.8	5.5	12.4	0.7	1140.0	440.0	-99.4	2.2
AFB-00	AFB_85	107.0	55.0	0.51	119.93	0.01	0.005	0.0019	0.0001	0.02	0.048	0.021	9.2	5.1	12.5	0.8	-180.0	590.0	26.2	-0.6
AFB-00	AFB_12	119.5	76.2	0.64	137.41	0.01	0.004	0.0019	0.0001	-0.32	0.058	0.017	13.7	4.1	12.5	0.7	130.0	470.0	-9.9	0.3

S1 Table. Zircon LA-ICP-MS U-Pb Isotopic Data and Ages for Smith et al., First U-Pb Zircon Ages from Ashfall Fossil Beds (AFB-00) and Grove Lake (GL-00), Nebraska, USA

						Corrected Isotopic Ratios							Ages (Ma)[h]							
Sample ID	Grain #[a]	U[b] [ppm]	Th[b] [ppm]	Th/U	eU[c] [ppm]	$^{207}Pb/^{235}U$[d]	±2s[e]	$^{206}Pb/^{238}U$[d]	±2s[e]	Rho[f]	$^{207}Pb/^{206}Pb$[g]	±2s[e]	$^{207}Pb/^{235}U$	±2s	$^{206}Pb/^{238}U$	±2s	$^{207}Pb/^{206}Pb$	±2s	Disc. %[k]	Wtd. Disc.[l]
AFB-00	AFB_148	112.0	63.2	0.56	126.85	0.01	0.005	0.0019	0.0001	-0.13	0.061	0.022	12.8	5.0	12.5	0.7	-50.0	520.0	-2.6	0.1
AFB-00	AFB_82	135.1	59.3	0.44	149.04	0.02	0.005	0.0019	0.0001	0.25	0.064	0.021	15.8	4.8	12.5	0.7	110.0	540.0	-26.6	0.7
AFB-00	AFB_5	98.1	49.5	0.50	109.73	0.04	0.008	0.0020	0.0001	0.45	0.145	0.031	34.4	8.0	12.6	0.8	1600.0	490.0	-172.6	2.7
AFB-00	AFB_43	69.1	35.5	0.51	77.44	0.02	0.008	0.0020	0.0001	0.17	0.065	0.031	18.2	7.9	12.6	0.8	-370.0	770.0	-44.1	0.7
AFB-00	AFB_123	93.3	55.8	0.60	106.41	0.01	0.006	0.0020	0.0002	-0.11	0.042	0.024	11.1	5.8	12.6	0.9	-480.0	660.0	12.2	-0.3
AFB-00	AFB_101	150.3	67.8	0.45	166.23	0.02	0.004	0.0020	0.0001	0.01	0.059	0.016	15.9	4.2	12.7	0.8	400.0	450.0	-25.0	0.8
AFB-00	AFB_33	51.7	39.5	0.76	60.98	0.08	0.015	0.0020	0.0002	0.41	0.304	0.056	80.0	13.0	12.8	1.4	3280.0	410.0	-525.0	5.2
AFB-00	AFB_1	114.2	69.8	0.61	130.60	0.04	0.005	0.0020	0.0001	-0.21	0.140	0.025	36.0	5.2	12.9	0.7	2030.0	320.0	-179.7	4.4
AFB-00	AFB_134	112.0	57.9	0.52	125.61	0.02	0.006	0.0020	0.0001	0.37	0.068	0.022	18.2	5.5	12.9	0.8	470.0	550.0	-40.6	1.0
AFB-00	AFB_62	121.5	78.6	0.65	139.97	0.02	0.007	0.0020	0.0001	0.04	0.053	0.022	15.3	6.8	13.0	0.9	100.0	630.0	-17.9	0.3
AFB-00	AFB_150	79.3	37.1	0.47	88.02	0.02	0.011	0.0020	0.0002	-0.20	0.093	0.043	23.0	11.0	13.0	1.0	880.0	870.0	-76.9	0.9
AFB-00	AFB_44	95.4	52.0	0.55	107.62	0.02	0.006	0.0020	0.0001	0.04	0.064	0.026	15.5	5.5	13.1	0.8	150.0	550.0	-18.4	0.4
AFB-00	AFB_130	124.0	71.5	0.58	140.80	0.02	0.005	0.0020	0.0001	0.06	0.066	0.021	15.2	5.0	13.1	0.8	-20.0	490.0	-15.9	0.4
AFB-00	AFB_60	166.0	90.9	0.55	187.36	0.03	0.005	0.0020	0.0001	0.23	0.093	0.018	25.2	4.8	13.1	0.8	1030.0	420.0	-91.9	2.5
AFB-00	AFB_46	101.3	53.0	0.52	113.76	0.04	0.007	0.0021	0.0001	0.29	0.155	0.025	38.5	6.4	13.3	0.8	2090.0	340.0	-190.1	3.9
AFB-00	AFB_78	761.0	143.5	0.19	794.72	0.01	0.001	0.0021	0.0001	-0.12	0.052	0.006	14.3	1.3	13.4	0.5	180.0	180.0	-7.0	0.7
AFB-00	AFB_63	137.8	65.6	0.48	153.22	0.05	0.009	0.0022	0.0002	0.55	0.153	0.031	44.3	9.0	13.9	1.0	2220.0	390.0	-219.6	3.4
AFB-00	AFB_69	94.2	40.6	0.43	103.74	0.05	0.007	0.0022	0.0002	0.06	0.191	0.028	53.1	7.3	14.0	1.0	2610.0	260.0	-278.5	5.4
AFB-00	AFB_103	157.0	101.7	0.65	180.90	0.06	0.015	0.0022	0.0002	0.40	0.199	0.049	56.0	15.0	14.1	1.1	2300.0	590.0	-297.2	2.8
AFB-00	AFB_74	148.7	95.2	0.64	171.07	0.02	0.007	0.0022	0.0001	0.21	0.074	0.025	18.6	6.7	14.2	0.8	440.0	570.0	-31.3	0.7
AFB-00	AFB_141	205.0	102.0	0.50	228.97	0.03	0.005	0.0022	0.0001	0.27	0.094	0.013	30.3	4.4	14.3	0.5	1480.0	280.0	-111.6	3.6
AFB-00	AFB_68	120.6	60.5	0.50	134.82	0.09	0.026	0.0025	0.0002	0.24	0.240	0.051	82.0	20.0	15.9	1.3	2520.0	460.0	-415.7	3.3
AFB-00	AFB_34	592.0	315.0	0.53	666.03	0.02	0.001	0.0025	0.0001	0.07	0.049	0.004	17.2	1.4	16.1	0.4	170.0	160.0	-7.0	0.8
AFB-00	AFB_81	183.7	129.7	0.71	214.18	0.09	0.011	0.0025	0.0001	0.40	0.270	0.028	89.8	9.9	16.3	0.8	3250.0	170.0	-450.9	7.4
AFB-00	AFB_106	90.8	35.7	0.39	99.19	0.10	0.019	0.0029	0.0002	0.33	0.272	0.046	98.0	17.0	18.8	1.3	3090.0	280.0	-421.3	4.7
AFB-00	AFB_138	153.5	92.2	0.60	175.17	0.16	0.016	0.0030	0.0002	0.56	0.402	0.030	152.0	13.0	19.1	1.1	3840.0	120.0	-695.8	10.2
AFB-00	AFB_39	100.8	57.9	0.57	114.41	0.21	0.029	0.0033	0.0002	0.67	0.415	0.047	186.0	25.0	21.1	1.5	3790.0	240.0	-781.5	6.6
AFB-00	AFB_41	430.0	148.1	0.34	464.80	0.03	0.002	0.0039	0.0001	0.03	0.048	0.004	26.4	2.1	25.2	0.6	120.0	140.0	-4.7	0.6
AFB-00	AFB_16	518.0	353.0	0.68	600.96	0.03	0.002	0.0043	0.0001	0.01	0.048	0.003	29.1	1.6	27.8	0.5	110.0	120.0	-4.6	0.8
AFB-00	AFB_31	227.0	151.0	0.67	262.49	0.03	0.003	0.0043	0.0001	0.06	0.052	0.006	30.6	3.1	27.9	0.8	240.0	200.0	-9.6	0.9
AFB-00	AFB_118	384.0	256.0	0.67	444.16	0.03	0.002	0.0053	0.0001	-0.03	0.045	0.003	32.5	2.1	34.3	0.8	-40.0	130.0	5.1	-0.8
AFB-00	AFB_90	216.0	155.0	0.72	252.43	0.04	0.005	0.0055	0.0002	0.23	0.057	0.006	42.4	4.7	35.0	1.2	470.0	210.0	-21.1	1.6
AFB-00	AFB_58	1210.0	1770.0	1.46	1625.95	0.04	0.002	0.0054	0.0001	0.30	0.051	0.002	37.2	1.7	35.0	0.7	207.0	92.0	-6.3	1.3
AFB-00	AFB_83	220.0	152.0	0.69	255.72	0.04	0.004	0.0055	0.0002	0.06	0.054	0.006	40.4	4.3	35.4	0.9	340.0	220.0	-14.2	1.2
AFB-00	AFB_7	178.1	203.9	1.14	226.02	0.04	0.004	0.0057	0.0002	0.03	0.046	0.005	35.4	3.5	36.9	0.9	20.0	180.0	4.0	-0.4
AFB-00	AFB_36	1528.0	390.0	0.26	1619.65	0.04	0.002	0.0060	0.0001	0.35	0.048	0.002	39.6	1.4	38.5	0.7	121.0	68.0	-3.0	0.8
AFB-00	AFB_87	515.0	142.0	0.28	548.37	0.04	0.002	0.0062	0.0001	0.10	0.047	0.002	40.0	2.0	39.9	0.7	67.0	99.0	-0.2	0.0
AFB-00	AFB_20	283.0	53.7	0.19	295.62	0.04	0.003	0.0064	0.0001	-0.23	0.046	0.004	40.4	3.2	41.4	0.8	30.0	160.0	2.3	-0.3
AFB-00	AFB_65	168.0	73.4	0.44	185.25	0.08	0.008	0.0117	0.0005	0.40	0.051	0.005	79.4	7.6	74.9	3.3	240.0	170.0	-6.0	0.6
AFB-00	AFB_125	235.7	94.4	0.40	257.88	0.08	0.004	0.0121	0.0003	-0.14	0.049	0.003	78.0	4.0	77.5	1.6	120.0	110.0	-0.6	0.1
AFB-00	AFB_54	169.0	55.2	0.33	181.97	0.10	0.006	0.0123	0.0005	0.15	0.059	0.004	94.2	5.7	78.6	2.8	520.0	150.0	-19.8	2.7
AFB-00	AFB_119	468.0	217.0	0.46	519.00	0.11	0.003	0.0168	0.0003	0.43	0.049	0.001	109.0	2.9	107.1	1.6	155.0	64.0	-1.8	0.7
AFB-00	AFB_51	221.7	123.9	0.56	250.82	1.03	0.110	0.0245	0.0009	0.82	0.295	0.026	691.0	58.0	156.1	5.6	3330.0	160.0	-342.7	9.2
AFB-00	AFB_145	245.0	382.0	1.56	334.77	0.18	0.009	0.0257	0.0004	0.06	0.052	0.003	169.6	7.6	163.6	2.7	233.0	97.0	-3.7	0.8
AFB-00	AFB_24	165.3	136.0	0.82	197.26	0.19	0.008	0.0273	0.0005	0.22	0.050	0.002	175.8	6.6	173.5	2.8	198.0	89.0	-1.3	0.3
AFB-00	AFB_126	218.0	173.9	0.80	258.87	0.22	0.008	0.0304	0.0006	0.43	0.051	0.002	199.0	6.4	192.9	4.0	234.0	71.0	-3.2	1.0
AFB-00	AFB_13	585.0	270.0	0.46	648.45	0.27	0.010	0.0367	0.0005	0.47	0.054	0.002	244.7	7.4	232.5	3.1	331.0	66.0	-5.2	1.6
AFB-00	AFB_136	436.0	248.0	0.57	494.28	6.17	0.530	0.0671	0.0048	0.99	0.674	0.018	1970.0	70.0	418.0	29.0	4677.0	42.0	-371.3	22.2
AFB-00	AFB_96	191.0	102.9	0.54	215.18	0.51	0.013	0.0678	0.0010	0.26	0.053	0.001	417.1	8.3	422.6	6.1	341.0	57.0	1.3	-0.7
AFB-00	AFB_48	165.0	123.1	0.75	193.93	0.57	0.018	0.0719	0.0016	0.18	0.059	0.002	460.0	12.0	447.4	9.5	551.0	83.0	-2.8	1.1
AFB-00	AFB_35	245.0	34.0	0.14	252.99	0.56	0.010	0.0727	0.0012	0.47	0.056	0.001	449.7	6.4	452.1	7.1	444.0	44.0	0.5	-0.4
AFB-00	AFB_124	146.0	50.0	0.34	157.75	0.79	0.036	0.0922	0.0033	0.71	0.061	0.002	587.0	20.0	568.0	20.0	621.0	56.0	-3.3	1.0
AFB-00	AFB_61	376.0	197.0	0.52	422.30	1.47	0.120	0.1091	0.0078	0.98	0.097	0.002	902.0	46.0	664.0	45.0	1549.0	37.0	-35.8	5.2

S1 Table. Zircon LA-ICP-MS U-Pb Isotopic Data and Ages for Smith et al., First U-Pb Zircon Ages from Ashfall Fossil Beds (AFB-00) and Grove Lake (GL-00), Nebraska, USA

						Corrected Isotopic Ratios							Ages (Ma)[h]							
Sample ID	Grain #[a]	U[b] [ppm]	Th[b] [ppm]	Th/U	eU[c] [ppm]	$^{207}Pb/^{235}U$[d]	±2s[e]	$^{206}Pb/^{238}U$[d]	±2s[e]	Rho[f]	$^{207}Pb/^{206}Pb$[g]	±2s[e]	$^{207}Pb/^{235}U$	±2s	$^{206}Pb/^{238}U$	±2s	$^{207}Pb/^{206}Pb$	±2s	Disc. %[k]	Wtd. Disc.[l]
AFB-00	AFB_23	116.7	41.8	0.36	126.52	1.66	0.034	0.1650	0.0025	0.36	0.074	0.002	995.0	13.0	984.0	14.0	1033.0	47.0	-1.1	3.5
AFB-00	AFB_22	83.2	81.8	0.98	102.42	1.82	0.058	0.1739	0.0034	0.29	0.076	0.002	1052.0	21.0	1033.0	19.0	1087.0	57.0	-1.8	2.8
AFB-00	AFB_10	232.0	2.1	0.01	232.49	1.79	0.040	0.1770	0.0026	0.47	0.074	0.002	1042.0	15.0	1050.0	14.0	1029.0	43.0	0.8	-1.5
AFB-00	AFB_79	672.0	17.4	0.03	676.09	2.48	0.075	0.1780	0.0058	0.91	0.101	0.002	1265.0	22.0	1054.0	32.0	1635.0	31.0	-20.0	18.2
AFB-00	AFB_11	87.7	61.0	0.70	102.04	1.86	0.038	0.1779	0.0027	0.35	0.075	0.002	1064.0	13.0	1055.0	15.0	1079.0	42.0	-0.9	1.6
AFB-00	AFB_59	319.0	154.1	0.48	355.21	1.96	0.040	0.1831	0.0031	0.68	0.078	0.001	1103.0	14.0	1085.0	17.0	1139.0	35.0	-1.7	3.2
AFB-00	AFB_115	106.3	129.2	1.22	136.66	2.00	0.040	0.1857	0.0026	0.56	0.078	0.002	1114.0	14.0	1098.0	14.0	1153.0	39.0	-1.5	3.9
AFB-00	AFB_121	32.9	18.7	0.57	37.29	1.98	0.085	0.1906	0.0036	0.19	0.078	0.003	1117.0	29.0	1124.0	19.0	1150.0	82.0	0.6	1.4
AFB-00	AFB_64	667.0	186.5	0.28	710.83	3.31	0.140	0.2043	0.0096	0.98	0.118	0.002	1480.0	34.0	1195.0	51.0	1922.0	26.0	-23.8	14.3
AFB-00	AFB_95	34.3	9.7	0.28	36.59	3.16	0.085	0.2332	0.0045	0.37	0.099	0.003	1450.0	21.0	1351.0	24.0	1587.0	53.0	-7.3	9.8
AFB-00	AFB_66	31.4	22.3	0.71	36.64	2.86	0.088	0.2381	0.0047	0.48	0.089	0.003	1384.0	23.0	1376.0	24.0	1404.0	56.0	-0.6	1.2
AFB-00	AFB_151	103.6	40.9	0.39	113.21	3.04	0.078	0.2446	0.0040	0.35	0.091	0.002	1416.0	20.0	1410.0	21.0	1444.0	49.0	-0.4	1.6
AFB-00	AFB_45	163.0	51.6	0.32	175.13	3.49	0.081	0.2693	0.0059	0.79	0.095	0.002	1525.0	18.0	1540.0	29.0	1527.0	34.0	1.0	-0.4
AFB-00	AFB_19	101.3	36.7	0.36	109.92	3.46	0.091	0.2740	0.0048	0.66	0.092	0.002	1516.0	21.0	1561.0	24.0	1466.0	40.0	2.9	-4.0
AFB-00	AFB_25	54.5	21.3	0.39	59.50	3.74	0.079	0.2759	0.0043	0.50	0.099	0.002	1579.0	17.0	1570.0	22.0	1596.0	41.0	-0.6	1.2
AFB-00	AFB_9	420.0	54.3	0.13	432.76	4.20	0.076	0.2933	0.0048	0.72	0.103	0.002	1671.0	15.0	1657.0	24.0	1682.0	28.0	-0.8	1.0
AFB-00	AFB_137	129.0	35.7	0.28	137.39	4.22	0.086	0.2948	0.0061	0.80	0.104	0.002	1675.0	17.0	1664.0	30.0	1699.0	31.0	-0.7	1.2
AFB-00	AFB_146	292.0	41.7	0.14	301.80	4.36	0.110	0.2952	0.0094	0.78	0.107	0.002	1704.0	21.0	1666.0	47.0	1748.0	37.0	-2.3	1.7
AFB-00	AFB_3	311.0	58.4	0.19	324.72	4.22	0.059	0.2966	0.0042	0.80	0.103	0.002	1680.0	12.0	1674.0	21.0	1677.0	26.0	-0.4	0.1
AFB-00	AFB_149	223.9	43.9	0.20	234.22	4.31	0.062	0.3001	0.0040	0.77	0.104	0.001	1694.0	12.0	1691.0	20.0	1705.0	26.0	-0.2	0.7
AFB-00	AFB_120	401.0	341.0	0.85	481.14	4.31	0.066	0.3002	0.0050	0.79	0.105	0.002	1696.0	13.0	1692.0	25.0	1713.0	27.0	-0.2	0.8
AFB-00	AFB_29	417.0	243.0	0.58	474.11	4.43	0.086	0.3012	0.0051	0.82	0.106	0.002	1718.0	16.0	1697.0	25.0	1725.0	27.0	-1.2	1.1
AFB-00	AFB_91	181.0	83.0	0.46	200.51	4.41	0.095	0.3023	0.0056	0.69	0.107	0.002	1713.0	18.0	1705.0	27.0	1741.0	36.0	-0.5	1.3
AFB-00	AFB_2	9.5	4.0	0.42	10.40	11.25	0.640	0.3081	0.0090	0.72	0.266	0.012	2538.0	51.0	1730.0	44.0	3295.0	70.0	-46.7	35.6
AFB-00	AFB_72	269.0	96.5	0.36	291.68	4.62	0.099	0.3101	0.0069	0.89	0.107	0.002	1752.0	18.0	1740.0	34.0	1753.0	28.0	-0.7	0.4
AFB-00	AFB_117	145.3	39.8	0.27	154.65	4.60	0.083	0.3106	0.0062	0.85	0.108	0.002	1749.0	15.0	1743.0	30.0	1757.0	28.0	-0.3	0.5
AFB-00	AFB_50	200.0	61.3	0.31	214.41	4.61	0.110	0.3120	0.0069	0.82	0.108	0.002	1758.0	19.0	1749.0	34.0	1759.0	32.0	-0.5	0.3
AFB-00	AFB_113	152.0	52.0	0.34	164.22	4.58	0.120	0.3137	0.0068	0.79	0.106	0.002	1745.0	21.0	1758.0	33.0	1726.0	40.0	0.7	-1.0
AFB-00	AFB_100	187.0	77.8	0.42	205.28	4.65	0.160	0.3130	0.0110	0.93	0.106	0.002	1760.0	30.0	1765.0	53.0	1733.0	33.0	0.3	-0.6
AFB-00	AFB_97	202.0	56.6	0.28	215.30	4.66	0.100	0.3182	0.0067	0.78	0.106	0.002	1759.0	18.0	1780.0	33.0	1726.0	35.0	1.2	-1.6
AFB-00	AFB_116	144.0	28.3	0.20	150.65	4.77	0.110	0.3217	0.0072	0.76	0.106	0.002	1780.0	19.0	1797.0	35.0	1734.0	34.0	0.9	-1.8
AFB-00	AFB_135	308.0	77.2	0.25	326.14	4.96	0.120	0.3264	0.0060	0.50	0.109	0.002	1811.0	21.0	1823.0	29.0	1776.0	34.0	0.7	-1.6
AFB-00	AFB_15	23.2	15.7	0.68	26.89	5.76	0.120	0.3479	0.0056	0.45	0.121	0.003	1939.0	18.0	1924.0	27.0	1965.0	39.0	-0.8	1.5
AFB-00	AFB_40	98.3	27.9	0.28	104.86	5.70	0.091	0.3556	0.0048	0.70	0.117	0.002	1930.0	14.0	1961.0	23.0	1907.0	27.0	1.6	-2.3
GL-00	GL_79	3,400.0	2,330.0	0.69	3947.55	0.01	0.000	0.0009	0.0000	0.19	0.047	0.003	5.5	0.4	5.5	0.2	110.0	120.0	0.0	0.0
GL-00	GL_27	236.0	140.7	0.60	269.06	0.01	0.003	0.0009	0.0001	0.21	0.071	0.023	9.3	2.5	5.8	0.5	280.0	510.0	-60.3	1.4
GL-00	GL_76	188.5	114.5	0.61	215.41	0.01	0.003	0.0009	0.0001	0.21	0.050	0.025	6.3	2.7	5.9	0.7	-130.0	640.0	-7.5	0.2
GL-00	GL_39	370.0	280.2	0.76	435.85	0.01	0.002	0.0009	0.0001	0.09	0.044	0.012	6.5	1.6	5.9	0.3	70.0	370.0	-10.4	0.4
GL-00	GL_50	452.0	313.1	0.69	525.58	0.01	0.002	0.0009	0.0001	0.03	0.058	0.015	7.2	1.9	5.9	0.4	290.0	460.0	-21.6	0.7
GL-00	GL_8	125.2	75.1	0.60	142.85	0.02	0.004	0.0009	0.0001	0.14	0.199	0.061	19.8	4.4	6.0	0.6	1250.0	710.0	-232.2	3.1
GL-00	GL_40	256.0	162.0	0.63	294.07	0.01	0.003	0.0009	0.0001	0.08	0.067	0.025	7.5	2.6	6.1	0.5	110.0	470.0	-23.6	0.6
GL-00	GL_28	357.0	243.8	0.68	414.29	0.01	0.002	0.0009	0.0001	0.01	0.067	0.014	9.3	1.9	6.1	0.4	580.0	380.0	-53.0	1.7
GL-00	GL_4	227.7	129.3	0.57	258.09	0.01	0.003	0.0009	0.0001	0.07	0.059	0.025	7.3	2.6	6.1	0.5	-120.0	520.0	-19.7	0.5
GL-00	GL_31	393.0	265.0	0.67	455.28	0.01	0.002	0.0009	0.0001	0.03	0.049	0.013	5.6	1.5	6.1	0.4	-80.0	370.0	8.2	-0.3
GL-00	GL_98	217.0	134.0	0.62	248.49	0.01	0.003	0.0009	0.0001	-0.12	0.063	0.035	6.2	3.0	6.1	0.6	-320.0	710.0	-1.6	0.0
GL-00	GL_55	292.9	174.9	0.60	334.00	0.02	0.003	0.0009	0.0001	0.20	0.116	0.021	15.3	2.5	6.1	0.4	1630.0	340.0	-150.4	3.7
GL-00	GL_9	198.6	123.2	0.62	227.55	0.01	0.003	0.0010	0.0001	0.16	0.110	0.039	10.6	3.3	6.1	0.5	160.0	610.0	-72.9	1.4
GL-00	GL_61	463.0	324.0	0.70	539.14	0.01	0.002	0.0010	0.0001	0.08	0.056	0.015	7.0	1.9	6.1	0.4	280.0	420.0	-14.2	0.5
GL-00	GL_49	448.0	250.0	0.56	506.75	0.01	0.002	0.0010	0.0001	0.15	0.054	0.013	6.4	1.7	6.1	0.4	50.0	410.0	-4.2	0.2
GL-00	GL_6	910.0	730.0	0.80	1081.55	0.01	0.002	0.0010	0.0001	-0.02	0.059	0.013	7.5	1.5	6.2	0.4	340.0	340.0	-22.0	0.9
GL-00	GL_84	457.0	309.0	0.68	529.62	0.01	0.002	0.0010	0.0001	0.03	0.061	0.016	7.3	1.9	6.2	0.5	280.0	420.0	-18.7	0.6
GL-00	GL_48	286.0	199.0	0.70	332.77	0.01	0.002	0.0010	0.0001	-0.01	0.060	0.018	7.4	2.2	6.2	0.5	250.0	460.0	-20.1	0.6
GL-00	GL_51	215.2	123.5	0.57	244.22	0.01	0.003	0.0010	0.0001	0.24	0.056	0.025	6.5	3.0	6.2	0.6	110.0	690.0	-5.5	0.1

S1 Table. Zircon LA-ICP-MS U-Pb Isotopic Data and Ages for Smith et al., First U-Pb Zircon Ages from Ashfall Fossil Beds (AFB-00) and Grove Lake (GL-00), Nebraska, USA

						Corrected Isotopic Ratios							Ages (Ma)[h]							
Sample ID	Grain #[a]	U[b] [ppm]	Th[b] [ppm]	Th/U	eU[c] [ppm]	$^{207}Pb/^{235}U$[d]	±2s[e]	$^{206}Pb/^{238}U$[d]	±2s[e]	Rho[f]	$^{207}Pb/^{206}Pb$[g]	±2s[e]	$^{207}Pb/^{235}U$	±2s	$^{206}Pb/^{238}U$	±2s	$^{207}Pb/^{206}Pb$	±2s	Disc. %[k]	Wtd. Disc.[l]
GL-00	GL_20	201.0	123.1	0.61	229.93	0.01	0.002	0.0010	0.0001	0.00	0.065	0.019	8.5	2.4	6.2	0.5	500.0	460.0	-37.8	1.0
GL-00	GL_53	202.0	115.0	0.57	229.03	0.01	0.003	0.0010	0.0001	-0.19	0.073	0.034	7.1	3.2	6.2	0.5	-10.0	630.0	-14.7	0.3
GL-00	GL_81	1,133.0	672.0	0.59	1290.92	0.01	0.001	0.0010	0.0000	0.09	0.050	0.006	6.7	0.8	6.2	0.3	230.0	210.0	-8.2	0.6
GL-00	GL_82	394.0	272.0	0.69	457.92	0.01	0.002	0.0010	0.0001	-0.12	0.052	0.012	6.6	1.5	6.2	0.4	30.0	370.0	-6.3	0.3
GL-00	GL_83	493.0	300.0	0.61	563.50	0.01	0.002	0.0010	0.0001	-0.24	0.058	0.015	6.3	1.6	6.2	0.4	40.0	400.0	-1.3	0.1
GL-00	GL_35	175.0	92.0	0.53	196.62	0.01	0.004	0.0010	0.0001	0.10	0.060	0.043	7.0	4.4	6.2	0.6	-1020.0	870.0	-12.4	0.2
GL-00	GL_65	293.0	193.0	0.66	338.36	0.01	0.003	0.0010	0.0001	-0.12	0.059	0.022	8.5	2.8	6.3	0.5	270.0	580.0	-35.8	0.8
GL-00	GL_102	1,080.0	800.0	0.74	1268.00	0.01	0.001	0.0010	0.0001	0.07	0.047	0.010	6.3	1.2	6.3	0.3	160.0	300.0	-0.6	0.0
GL-00	GL_57	131.3	61.9	0.47	145.85	0.01	0.004	0.0010	0.0001	0.07	0.047	0.044	6.8	3.9	6.3	0.7	-1500.0	1200.0	-8.5	0.1
GL-00	GL_67	324.0	220.0	0.68	375.70	0.01	0.002	0.0010	0.0001	-0.08	0.062	0.017	7.7	2.0	6.3	0.5	270.0	440.0	-22.4	0.7
GL-00	GL_75	252.0	157.4	0.62	288.99	0.01	0.002	0.0010	0.0001	-0.04	0.041	0.018	5.3	2.0	6.3	0.5	-170.0	500.0	16.0	-0.5
GL-00	GL_5	456.0	259.0	0.57	516.87	0.01	0.003	0.0010	0.0001	0.36	0.051	0.020	7.1	2.8	6.4	0.5	170.0	590.0	-11.6	0.3
GL-00	GL_22	335.0	215.0	0.64	385.53	0.01	0.002	0.0010	0.0001	-0.01	0.075	0.019	9.7	2.0	6.4	0.5	510.0	380.0	-52.3	1.7
GL-00	GL_77	328.0	221.9	0.68	380.15	0.01	0.002	0.0010	0.0001	0.07	0.043	0.015	6.3	2.0	6.4	0.4	-110.0	440.0	1.3	0.0
GL-00	GL_96	288.0	178.1	0.62	329.85	0.01	0.003	0.0010	0.0001	-0.04	0.069	0.021	9.7	2.7	6.4	0.5	630.0	500.0	-51.8	1.2
GL-00	GL_13	90.9	59.1	0.65	104.79	0.01	0.008	0.0010	0.0002	0.02	0.140	0.170	8.3	7.9	6.4	0.9	-1500.0	1700.0	-29.7	0.2
GL-00	GL_10	395.0	259.0	0.66	455.87	0.01	0.001	0.0010	0.0001	0.15	0.050	0.011	6.7	1.4	6.4	0.4	20.0	350.0	-4.5	0.2
GL-00	GL_42	272.0	188.0	0.69	316.18	0.01	0.002	0.0010	0.0001	0.18	0.067	0.020	7.7	2.4	6.4	0.4	300.0	480.0	-20.1	0.5
GL-00	GL_100	342.0	251.0	0.73	400.99	0.01	0.002	0.0010	0.0001	0.04	0.048	0.017	7.2	1.9	6.4	0.4	100.0	450.0	-12.3	0.4
GL-00	GL_47	1,550.0	1,024.0	0.66	1790.64	0.01	0.001	0.0010	0.0000	0.18	0.044	0.005	6.2	0.7	6.4	0.3	-20.0	180.0	3.4	-0.3
GL-00	GL_92	375.0	239.0	0.64	431.17	0.01	0.002	0.0010	0.0001	0.07	0.055	0.012	7.4	1.6	6.4	0.4	280.0	360.0	-15.3	0.6
GL-00	GL_15	173.0	99.0	0.57	196.27	0.01	0.003	0.0010	0.0001	0.00	0.081	0.031	9.2	3.2	6.4	0.6	290.0	680.0	-43.1	0.9
GL-00	GL_62	177.0	88.5	0.50	197.80	0.01	0.003	0.0010	0.0001	-0.16	0.096	0.036	10.2	3.3	6.4	0.6	500.0	600.0	-58.6	1.1
GL-00	GL_2	262.0	156.0	0.60	298.66	0.01	0.003	0.0010	0.0001	-0.14	0.073	0.026	8.5	3.0	6.4	0.4	340.0	590.0	-32.0	0.7
GL-00	GL_32	397.0	285.2	0.72	464.02	0.01	0.003	0.0010	0.0001	0.01	0.061	0.021	8.1	2.6	6.4	0.4	-80.0	400.0	-25.8	0.6
GL-00	GL_89	264.0	148.0	0.56	298.78	0.01	0.002	0.0010	0.0001	0.16	0.063	0.023	8.1	2.4	6.4	0.5	-120.0	530.0	-25.8	0.7
GL-00	GL_33	294.5	186.7	0.63	338.37	0.01	0.002	0.0010	0.0001	0.20	0.094	0.017	11.6	2.1	6.5	0.4	940.0	380.0	-79.8	2.5
GL-00	GL_44	316.0	196.0	0.62	362.06	0.01	0.002	0.0010	0.0001	-0.10	0.062	0.021	8.4	2.3	6.5	0.6	600.0	540.0	-30.2	0.8
GL-00	GL_78	352.0	221.0	0.63	403.94	0.01	0.002	0.0010	0.0001	-0.06	0.044	0.017	5.7	2.0	6.5	0.5	-180.0	420.0	11.6	-0.4
GL-00	GL_11	282.0	209.0	0.74	331.12	0.01	0.002	0.0010	0.0001	-0.19	0.065	0.020	8.0	2.2	6.5	0.5	190.0	470.0	-23.8	0.7
GL-00	GL_24	177.0	92.0	0.52	198.62	0.01	0.003	0.0010	0.0001	-0.15	0.079	0.047	7.0	3.1	6.5	0.7	0.0	730.0	-7.9	0.2
GL-00	GL_85	1,256.0	1,200.0	0.96	1538.00	0.01	0.001	0.0010	0.0000	0.11	0.051	0.006	7.1	0.8	6.5	0.3	170.0	180.0	-9.2	0.8
GL-00	GL_21	84.4	51.9	0.61	96.60	0.00	0.006	0.0010	0.0001	0.06	0.075	0.072	4.0	6.5	6.5	0.8	-2100.0	1700.0	38.6	-0.4
GL-00	GL_29	265.0	156.0	0.59	301.66	0.01	0.003	0.0010	0.0001	0.09	0.081	0.025	10.0	2.8	6.5	0.6	490.0	540.0	-53.4	1.2
GL-00	GL_64	88.4	48.0	0.54	99.68	0.01	0.006	0.0010	0.0001	0.08	0.160	0.110	13.7	5.8	6.5	0.9	-700.0	1900.0	-109.5	1.2
GL-00	GL_34	312.3	209.6	0.67	361.56	0.01	0.002	0.0010	0.0001	0.19	0.053	0.013	7.0	1.7	6.6	0.4	140.0	370.0	-6.9	0.3
GL-00	GL_45	237.0	144.0	0.61	270.84	0.01	0.002	0.0010	0.0001	0.16	0.055	0.017	7.2	2.3	6.6	0.5	10.0	460.0	-9.9	0.3
GL-00	GL_71	321.0	170.5	0.53	361.07	0.01	0.003	0.0010	0.0001	-0.10	0.050	0.022	5.8	2.5	6.6	0.6	-340.0	590.0	11.7	-0.3
GL-00	GL_43	226.0	140.0	0.62	258.90	0.01	0.003	0.0010	0.0001	0.13	0.051	0.021	6.9	2.7	6.6	0.5	10.0	570.0	-4.7	0.1
GL-00	GL_59	317.0	206.0	0.65	365.41	0.01	0.003	0.0010	0.0001	0.11	0.067	0.026	8.5	3.2	6.6	0.6	-10.0	620.0	-28.8	0.6
GL-00	GL_66	329.0	212.0	0.64	378.82	0.01	0.002	0.0010	0.0001	0.07	0.078	0.015	10.4	1.9	6.6	0.4	780.0	380.0	-57.6	2.0
GL-00	GL_60	324.9	226.6	0.70	378.15	0.01	0.002	0.0010	0.0001	-0.28	0.063	0.016	7.2	1.8	6.6	0.5	330.0	420.0	-8.8	0.3
GL-00	GL_52	336.0	262.6	0.78	397.71	0.01	0.002	0.0010	0.0001	0.10	0.053	0.013	7.5	1.8	6.7	0.5	190.0	390.0	-12.8	0.5
GL-00	GL_26	305.0	195.0	0.64	350.83	0.01	0.003	0.0010	0.0001	-0.01	0.058	0.026	8.5	2.5	6.7	0.5	-40.0	460.0	-26.9	0.7
GL-00	GL_73	221.8	128.3	0.58	251.95	0.01	0.003	0.0010	0.0001	-0.08	0.057	0.022	6.1	2.7	6.7	0.5	140.0	570.0	9.2	-0.2
GL-00	GL_86	267.0	161.0	0.60	304.84	0.01	0.002	0.0010	0.0001	0.08	0.063	0.021	8.9	2.5	6.7	0.5	-120.0	550.0	-32.0	0.9
GL-00	GL_54	267.0	174.8	0.65	308.08	0.01	0.005	0.0010	0.0001	0.14	0.057	0.032	8.7	4.6	6.8	0.6	-110.0	580.0	-28.7	0.4
GL-00	GL_99	274.0	236.0	0.86	329.46	0.01	0.004	0.0011	0.0001	0.02	0.073	0.026	9.6	3.7	6.8	0.6	420.0	720.0	-41.8	0.8
GL-00	GL_69	280.0	185.0	0.66	323.48	0.01	0.003	0.0011	0.0001	0.19	0.066	0.022	9.0	2.7	6.8	0.6	230.0	590.0	-32.7	0.8
GL-00	GL_18	283.0	199.5	0.70	329.88	0.01	0.002	0.0011	0.0001	-0.07	0.051	0.016	6.6	1.8	6.8	0.4	-50.0	420.0	2.8	-0.1
GL-00	GL_12	274.0	160.8	0.59	311.79	0.01	0.003	0.0011	0.0001	-0.03	0.066	0.029	8.2	2.8	6.8	0.5	-300.0	560.0	-20.1	0.5
GL-00	GL_93	310.0	262.0	0.85	371.57	0.02	0.003	0.0011	0.0001	0.13	0.137	0.022	19.4	3.2	6.8	0.5	2010.0	310.0	-184.0	3.9
GL-00	GL_70	175.6	85.7	0.49	195.74	0.01	0.005	0.0011	0.0001	0.23	0.081	0.037	10.7	4.6	6.9	0.8	600.0	830.0	-56.0	0.8

S1 Table. Zircon LA-ICP-MS U-Pb Isotopic Data and Ages for Smith et al., First U-Pb Zircon Ages from Ashfall Fossil Beds (AFB-00) and Grove Lake (GL-00), Nebraska, USA

						Corrected Isotopic Ratios							Ages (Ma)[h]							
Sample ID	Grain #[a]	U[b] [ppm]	Th[b] [ppm]	Th/U	eU[c] [ppm]	$^{207}Pb/^{235}U$[d]	±2s[e]	$^{206}Pb/^{238}U$[d]	±2s[e]	Rho[f]	$^{207}Pb/^{206}Pb$[g]	±2s[e]	$^{207}Pb/^{235}U$	±2s	$^{206}Pb/^{238}U$	±2s	$^{207}Pb/^{206}Pb$	±2s	Disc. %[k]	Wtd. Disc.[l]
GL-00	GL_1	129.8	44.1	0.34	140.16	0.01	0.005	0.0011	0.0001	0.02	0.100	0.044	10.0	5.3	6.9	0.6	60.0	860.0	-45.6	0.6
GL-00	GL_90	202.0	102.7	0.51	226.13	0.02	0.004	0.0011	0.0001	0.13	0.139	0.031	18.1	4.0	7.0	0.7	1600.0	470.0	-160.1	2.8
GL-00	GL_72	226.0	118.0	0.52	253.73	0.01	0.002	0.0011	0.0001	-0.06	0.064	0.018	7.6	1.9	7.0	0.5	280.0	450.0	-8.6	0.3
GL-00	GL_95	421.0	283.6	0.67	487.65	0.02	0.004	0.0011	0.0001	0.02	0.146	0.029	20.7	4.0	7.1	0.6	1890.0	490.0	-192.4	3.4
GL-00	GL_17	142.0	77.3	0.54	160.17	0.01	0.004	0.0011	0.0001	-0.02	0.120	0.130	12.3	3.9	7.2	0.8	-150.0	970.0	-71.5	1.3
GL-00	GL_91	387.0	269.0	0.70	450.22	0.01	0.003	0.0011	0.0001	0.13	0.069	0.020	9.7	3.0	7.2	0.4	670.0	500.0	-35.3	0.8
GL-00	GL_56	11.8	3.8	0.32	12.70	0.03	0.043	0.0011	0.0006	0.14	-0.270	0.300	32.0	43.0	7.2	3.9	-9300.0	5300.0	-344.4	0.6
GL-00	GL_103	185.0	119.0	0.64	212.97	0.01	0.004	0.0011	0.0001	-0.25	0.058	0.034	7.0	4.1	7.3	0.8	0.0	770.0	4.0	-0.1
GL-00	GL_97	115.4	47.6	0.41	126.59	0.01	0.005	0.0012	0.0001	0.03	0.066	0.046	9.5	4.8	7.5	0.8	-540.0	880.0	-27.5	0.4
GL-00	GL_38	945.0	706.0	0.75	1110.91	0.03	0.004	0.0012	0.0001	0.47	0.173	0.018	27.8	3.4	7.6	0.4	2500.0	180.0	-266.3	5.9
GL-00	GL_88	130.9	65.8	0.50	146.36	0.02	0.005	0.0012	0.0001	0.17	0.139	0.033	22.7	5.1	7.7	0.7	1540.0	530.0	-193.3	2.9
GL-00	GL_63	134.8	95.3	0.71	157.20	0.02	0.006	0.0012	0.0001	0.27	0.161	0.045	23.8	6.2	7.8	0.8	1530.0	640.0	-206.7	2.6
GL-00	GL_94	103.0	50.6	0.49	114.89	0.02	0.009	0.0013	0.0002	0.15	0.153	0.098	16.4	8.7	8.6	1.0	400.0	1100.0	-90.9	0.9
GL-00	GL_46	158.0	103.2	0.65	182.25	0.04	0.006	0.0014	0.0001	-0.08	0.222	0.061	41.8	6.2	8.7	0.8	2480.0	390.0	-380.5	5.3
GL-00	GL_101	191.1	107.0	0.56	216.25	0.06	0.009	0.0014	0.0001	0.58	0.274	0.040	57.4	8.9	8.8	0.8	2930.0	360.0	-551.5	5.5
GL-00	GL_68	759.0	601.0	0.79	900.24	0.04	0.003	0.0014	0.0001	0.16	0.196	0.015	35.3	2.6	8.8	0.4	2750.0	140.0	-299.3	10.2
GL-00	GL_36	111.2	64.6	0.58	126.38	0.04	0.011	0.0014	0.0002	0.05	0.190	0.130	40.0	10.0	9.1	1.1	1760.0	960.0	-339.6	3.1
GL-00	GL_80	132.8	82.8	0.62	152.26	0.05	0.007	0.0015	0.0001	0.24	0.234	0.039	46.1	6.8	9.6	0.8	2690.0	350.0	-379.7	5.4
GL-00	GL_74	166.0	100.9	0.61	189.71	0.06	0.008	0.0015	0.0001	-0.13	0.328	0.048	61.3	7.1	9.7	0.7	3160.0	360.0	-532.0	7.3
GL-00	GL_14	147.8	87.1	0.59	168.27	0.09	0.010	0.0017	0.0001	0.06	0.432	0.053	88.4	8.8	11.1	0.9	3790.0	200.0	-695.0	8.8
GL-00	GL_87	151.2	59.1	0.39	165.09	0.12	0.013	0.0021	0.0002	0.13	0.446	0.063	112.0	12.0	13.2	1.3	3960.0	210.0	-748.5	8.2
GL-00	GL_16	8.1	2.5	0.31	8.65	0.13	0.069	0.0029	0.0012	0.12	-0.050	0.320	114.0	65.0	18.5	7.5	-10200.0	7700.0	-516.2	1.5
GL-00	GL_37	11.2	2.9	0.26	11.86	0.31	0.078	0.0031	0.0009	0.12	0.900	0.430	264.0	55.0	20.0	5.8	1100.0	4500.0	-1220.0	4.4
GL-00	GL_19	235.0	137.6	0.59	267.34	0.03	0.004	0.0041	0.0002	0.12	0.049	0.007	27.0	3.6	26.3	1.1	240.0	250.0	-2.5	0.2
GL-00	GL_30	2.9	0.6	0.19	3.05	0.02	0.220	0.0067	0.0038	-0.12	0.110	0.200	90.0	200.0	42.0	24.0	-7700.0	4200.0	-114.3	0.2
GL-00	GL_23	19.5	13.4	0.69	22.65	2.79	0.310	0.0242	0.0028	0.82	0.822	0.057	1316.0	93.0	153.0	18.0	5000.0	140.0	-760.1	12.5
GL-00	GL_7	23.1	16.0	0.69	26.86	2.71	0.680	0.0287	0.0073	0.90	0.784	0.064	1200.0	160.0	170.0	41.0	4920.0	150.0	-605.9	6.4
GL-00	GL_41	129.8	51.7	0.40	141.95	1.75	0.067	0.1707	0.0052	0.56	0.074	0.002	1026.0	24.0	1015.0	28.0	1035.0	50.0	-1.1	0.7
GL-00	GL_58	77.0	35.9	0.47	85.44	1.92	0.082	0.1812	0.0060	0.52	0.077	0.002	1086.0	29.0	1073.0	33.0	1143.0	57.0	-1.2	2.1
GL-00	GL_25	69.9	34.4	0.49	77.98	2.06	0.077	0.1943	0.0060	0.33	0.077	0.002	1134.0	25.0	1144.0	33.0	1116.0	57.0	0.9	-0.8

[a] Samples are sorted from youngest $^{206}Pb/^{238}U$-age to oldest; Highlighted rows indicate youngest concordant analyses used to calculate depositional ages

[b] U and Th concentrations and Th/U ratios calculated relative to the GJ-1 zircon standard ID-TIMS values using 287 ± 76 ppm for U and 8.4 ± 2.6 ppm for Th (Jackson et al., 2004)

[c] Equivalent U defined by the equation: eU = U ppm + 0.235*Th ppm

[d] Corrected for U-Pb fractionation and background and normalized to the GJ-1 zircon standard ID-TIMS values: $^{207}Pb/^{235}U$ = 0.8093 ± 0.0009 and $^{206}Pb/^{238}U$ = 0.09761 ± 0.00011 (Jackson et al., 2004)

[e] Propagated uncertainty of internal uncertainties (2 SE) and within run reproducibility of GJ-1 (2 SE)

[f] Uncertainty correlation between $^{206}Pb/^{238}U$ and $^{207}Pb/^{235}U$ uncertainties

[g] Corrected for background and Pb isotopic fractionation using the GJ-1 zircon standard ID-TIMS value: $^{207}Pb/^{206}Pb$ = 0.06014 ± 0.00001 (Jackson et al., 2004)

[h] U-Pb ages calculated relative to the weighted mean $^{206}Pb/^{238}U$ age of 600.4 ±0.65 Ma for the GJ-1 zircon reference material (Jackson et al., 2004)

[i] Percent Discordance defined as $(1-(^{207}Pb/^{235}U_{age} / ^{206}Pb/^{238}U_{age}))*100$

[j] Uncertainty weighted age difference defined as $(^{207}Pb/^{235}U_{age} - ^{206}Pb/^{238}U_{age})/(^{207}Pb/^{235}U_{2\sigma\ age\ uncertainty})$ for grains with $^{206}Pb/^{238}U$ ages <900 Ma and $(^{207}Pb/^{206}Pb_{age} - ^{206}Pb/^{238}U_{age})/(^{206}Pb/^{238}U_{2\sigma\ age\ uncertainty})$ for grains with $^{206}Pb/^{238}U$ ages ≥900 Ma

Jackson, S.E., Pearson, N.J., Griffin, W.L., and Belousova, E.A., 2004, The application of laser ablation-inductively coupled plasma-mass spectrometry to in situ U–Pb zircon geochronology: Chemical Geology, v. 211, p. 47-69.
