# Supplementary material for: First U-Pb zircon ages for late Miocene Ashfall Konservat-Lagerstätte and Grove Lake ashes from eastern Great Plains, USA
Source: PLoS One. 2018 Nov 8;13(11):e0207103. doi: 10.1371/journal.pone.0207103 (PMC6224108; doi:10.1371/journal.pone.0207103)
Supplement: S2 Table — (PDF) [file pone.0207103.s002.pdf]

S2 Table. Supervolcano Eruption Data; Smith et al. The first U-Pb zircon ages for the Ashfall Fossil Beds

Age (Ma)	Unit[a]	State	Volcanic Field	Estimated Volume (km^3)[b]	Reference
4.45	Kilgore Tuff	Idaho	Heise	1800	Morgan and McIntosh (2005)
5.51	Conant Creek Tuff	Idaho	Heise	300	Morgan and McIntosh (2005)
6.27	Walcott Tuff	Idaho	Heise	750	Morgan and McIntosh (2005)
6.62	Blacktail Creek Tuff	Idaho	Heise	1500	Morgan and McIntosh (2005)
7.02	Cub River Tuff	Idaho	Picabo	1000	Perkins and Nash (2002)
7.49	Faust Tuff	Idaho	Picabo	1000	Perkins and Nash (2002)
7.90	Rush Valley Tuff	Idaho	Picabo	1000	Perkins and Nash (2002)
8.30	Inkom Tuff	Idaho	Picabo	1000	Perkins and Nash (2002)
9.16	McMullen Creek Tuff	Idaho	Twin Falls	500	Perkins and Nash (2002)
9.41	Lonergan Creek Tuff	Idaho	Twin Falls	500	Perkins and Nash (2002)
9.52	Opal Canyon 6 Tuff	Idaho	Twin Falls	500	Perkins and Nash (2002)
10.25	Rawlins Tuff	Idaho	Twin Falls	500	Perkins and Nash (2002)
10.45	Cougar Point Tuff XV	Idaho	Bruneau-Jarbidge	500	Bonnichsen et al. (2008)
10.75	Cougar Point Tuff XIII	Idaho	Bruneau-Jarbidge	1000	Bonnichsen et al. (2008)
11.22	Cougar Point Tuff XI	Idaho	Bruneau-Jarbidge	1000	Bonnichsen et al. (2008)
11.59	Cougar Point Tuff IX	Idaho	Bruneau-Jarbidge	500	Bonnichsen et al. (2008)
11.81	Cougar Point Tuff VII	Idaho	Bruneau-Jarbidge	750	Bonnichsen et al. (2008)
11.93	Ibex Hollow Tuff	Idaho	Bruneau-Jarbidge	500	Perkins and Nash (2002)
12.67	Cougar Point Tuff III	Idaho	Bruneau-Jarbidge	500	Bonnichsen et al. (2008)
13.5	Hurlbut Tuff	Idaho	Owyhee-Humboldt	950	Perkins and Nash (2002)
13.80	Swisher Mountain Tuff	Idaho	Owyhee-Humboldt	1430	Ekren et al. (1984)

[a] Ash-flow tuffs geochemically correlated with airfall deposits on the Great Plains (from Figure 5 in Perkins and Nash, 2002).
[b] When possible, eruption volumes are from estimated ashfall volumes reported in cited references.

Bonnichsen B, Leeman WP, Honjo N, McIntosh WC, Godchaux MM (2008) Miocene silicic volcanism in southwestern Idaho: Geochronology, geochemistry, and evolution of the central Snake River Plain. Bulletin of Volcanology 70: 315–342.

Ekren EB, McIntyre DH, Bennett EH (1984) High-temperature, large-volume, lavalike ash-flow tuffs without calderas in southwestern Idaho. U.S. Geological Survey Professional Paper 1272: 76 p.

Morgan L A, McIntosh W C (2005) Timing and development of the Heise volcanic field, Snake River Plain, Idaho, western USA. Geological Society of America, Bulletin 117: 288-306.

Perkins, M.E., and Nash, B.P., 2002, Explosive silicic volcanism of the Yellowstone Hotspot: the ash fall tuff record: Geological Society of America, Bulletin, v. 114, p. 367–381.
